# Acetylation of Mitochondrial Proteins in the Heart: The Role of SIRT3

**DOI:** 10.3389/fphys.2018.01094

**Published:** 2018-08-07

**Authors:** Rebecca M. Parodi-Rullán, Xavier R. Chapa-Dubocq, Sabzali Javadov

**Affiliations:** Department of Physiology, University of Puerto Rico School of Medicine, San Juan, PR, United States

**Keywords:** mitochondria, protein acetylation, sirtuins, SIRT3, cardiac diseases, cardioprotection

## Abstract

A growing number of studies have demonstrated the role of post-translational modifications of proteins, particularly acetylation, in human diseases including neurodegenerative and cardiovascular diseases, diabetes, cancer, and in aging. Acetylation of mitochondrial proteins has been shown to be involved in the pathogenesis of cardiac diseases such as myocardial infarction (ischemia-reperfusion) and heart failure. Indeed, over 60% of mitochondrial proteins contain acetylation sites, and most of these proteins are involved in mitochondrial bioenergetics. Mitochondrial non-enzymatic acetylation is enabled by acetyl-coenzyme A abundance and serves as the primary pathway of acetylation in mitochondria. Hence, regulation of enzymatic deacetylation becomes the most important mechanism to control acetylation/deacetylation of mitochondrial proteins. Acetylation/deacetylation of mitochondrial proteins has been regarded as a key regulator of mitochondrial metabolism and function. Proteins are deacetylated by NAD^+^-dependent deacetylases known as sirtuins (SIRTs). Among seven sirtuin isoforms, only SIRT3, SIRT4, and SIRT5 are localized in the mitochondria. SIRT3 is the main mitochondrial sirtuin which plays a key role in maintaining metabolic and redox balance in the mitochondria under physiological and pathological conditions. SIRT3 regulates the enzymatic activity of proteins involved in fatty acid oxidation, tricarboxylic acid cycle, electron transport chain, and oxidative phosphorylation. Although many enzymes have been identified as targets for SIRT3, cardiac-specific SIRT3 effects and regulations could differ from those in non-cardiac tissues. Therefore, it is important to elucidate the contribution of SIRT3 and mitochondrial protein acetylation/deacetylation in mitochondrial metabolism and cardiac dysfunction. Here, we summarize previous studies and provide a comprehensive analysis of the role of SIRT3 in mitochondria metabolism and bioenergetics under physiological conditions and in cardiac diseases. In addition, the review discusses mitochondrial protein acetylation as a potential target for cardioprotection.

## Introduction

Post-translational modifications involve the covalent modification of a protein after it has been translated in order to change the enzymatic activity, alter protein–protein interactions, and mediate protein stability (Glozak et al., 2005). For over 50 years, acetylation has been known to play a role in the regulation of nuclear protein transcription. In 1997, the non-histone protein p53 was the first to be reported to be acetylated (Gu and Roeder, 1997). Since the discovery of non-histone protein acetylation, a growing number of studies have revealed that the activity of cytosolic and mitochondrial proteins can be regulated by acetylation.

Several PTMs such as phosphorylation, ubiquitination, sumoylation, and acetylation have been identified in mammalian cells. Acetylation involves the transfer of the acetyl group of acetyl-CoA to a lysine residue in a protein; therefore resulting in the disruption of the positively charged lysine into a non-charged residue. Two types of protein acetylation have been described: non-reversible N-terminal acetylation which occurs co-translationally, and reversible post-translational acetylation of a 𝜀-amino lysine residue. Deacetylation is regulated by four classes of histone deacetylases (HDACs). Sirtuins (SIRTs; silent mating type information regulation two homologs) are class III HDACs, which require NAD^+^ as a cofactor. In contrast to class I and class II HDACs that are zinc-dependent and sensitive to trichostatin A inhibition (Gray and Ekström, 2001), sirtuins are not sensitive to trichostatin A. In mammals, a total of seven sirtuins isoforms are found, SIRT1-7. Although all sirtuins are NAD^+^-dependent, they vary in subcellular localization, PTM types, and substrate affinity. SIRT1, SIRT6, and SIRT7 are the main nuclear sirtuins whereas SIRT2 localizes in both, the cytoplasm and nucleus. SIRT3, SIRT4, and SIRT5 are the mitochondrial sirtuins responsible for the regulation of protein acetylation. The main characteristics of sirtuins are given in **Table 1**. In this review, we summarize and discuss previous studies on the role of mitochondrial sirtuins, particularly SIRT3, in the regulation of mitochondrial metabolism and function in the heart. In addition, we will discuss the role of mitochondrial protein acetylation in the pathogenesis of cardiac diseases including MI, IR, cardiac hypertrophy, and HF. Finally, we will describe potential therapeutic strategies that are currently under development to target mitochondrial protein acetylation for treatment of cardiac diseases.

**Table 1:** Main functional and metabolic properties of sirtuins in the heart.

Sirtuins	Localization	Enzymatic activity	Role in cell metabolism	Role in cardiac diseases
**SIRT1**	Nucleus (Luo et al., 2001; Vaziri et al., 2001; Michishita et al., 2005), cytosol (Tanno et al., 2007)	Deacetylase (Luo et al., 2001; Vaziri et al., 2001)	Gene expression (Alcendor et al., 2007), apoptosis (Luo et al., 2001)	cardiac dysfunction and cell death (Alcendor et al., 2007), hypertrophy (Planavila et al., 2011), ischemic preconditioning (Nadtochiy et al., 2011)
**SIRT2**	Nucleus (Vaquero et al., 2006), cytoplasm (North et al., 2003), mitochondria (Liu et al., 2017)	Deacetylase (North et al., 2003), demyristoylase (Teng et al., 2015)	Cell cycle (Dryden et al., 2003; Bae et al., 2004), glucose metabolism (Arora and Dey, 2014; Watanabe et al., 2017)	Cardiac hypertrophy (Tang et al., 2017), hypoxia-reoxygenation (Lynn et al., 2008)
**SIRT3**	Mitochondria (Lombard et al., 2007)	Deacetylase (Lombard et al., 2007; Ahn et al., 2008)	Mitochondrial bioenergetics and metabolism (Hirschey et al., 2010; Finley et al., 2011; Fan et al., 2014; Dittenhafer-Reed et al., 2015; Koentges et al., 2015)	Ischemia-reperfusion (Porter et al., 2014; Parodi-Rullán et al., 2017), hypertrophy (Sundaresan et al., 2009; Pillai et al., 2010; Chen et al., 2015), heart failure (Grillon et al., 2012)
**SIRT4**	Mitochondria (Michishita et al., 2005)	ADP-ribosyltransferase (Ahuja et al., 2007)	Lipid metabolism (Nasrin et al., 2010; Laurent et al., 2013)	Hypertrophy (Luo et al., 2017), hypoxia (Liu et al., 2013a)
**SIRT5**	Mitochondria (Michishita et al., 2005)	Desuccinylase (Rardin et al., 2013a; Boylston et al., 2015), demalonylase (Du et al., 2011), deglutarylase (Tan et al., 2014)	Mitochondrial metabolism (Nakagawa et al., 2009; Park et al., 2013; Rardin et al., 2013a; Nishida et al., 2015)	Cardiac oxidative stress (Liu et al., 2013b), ischemia-reperfusion (Boylston et al., 2015)
**SIRT6**	Nucleus (Liszt et al., 2005; Michishita et al., 2005; Mostoslavsky et al., 2006; Sundaresan et al., 2012)	Deacetylase, (Michishita et al., 2008, 2009; Yang et al., 2009), ADP-ribosyltransferase (Liszt et al., 2005)	Histone deacetylation (Michishita et al., 2008, 2009; Yang et al., 2009)	Hypertrophy (Sundaresan et al., 2012)
**SIRT7**	Nucleus/nucleolus (Michishita et al., 2005), cytoplasm (Kiran et al., 2013)	Deacetylase (Tong et al., 2016), deacylase (Tong et al., 2017)	Regulation of nuclear-encoded mitochondrial genes (Ryu et al., 2014)	Cardiac hypertrophy, fibrosis, apoptosis (Vakhrusheva et al., 2008), angiogenesis and scar formation (Araki et al., 2015)

## The Role of Sirtuins in the Heart

### Cytoplasmic Sirtuins (SIRT2)

In the cell, SIRT2 is localized in the cytoplasm (North et al., 2003), and it is in contact with chromatin in the nucleus (Vaquero et al., 2006). In addition to its deacetylase activity, SIRT2 possesses demyristoylase activity *in vitro* (Teng et al., 2015). It is most abundant in the central nervous system (Maxwell et al., 2011) and has important roles in regulating tubulin acetylation (North et al., 2003), cell cycle (Dryden et al., 2003; Bae et al., 2004), and glucose metabolism (Arora and Dey, 2014; Watanabe et al., 2017). The myocardial SIRT2 expression is reduced during cardiac hypertrophy (Tang et al., 2017). The role of SIRT2 in mammalian cell metabolism is discussed elsewhere (Gomes et al., 2015; Bindu et al., 2016).

### Nuclear Sirtuins (SIRT1, SIRT6, and SIRT7)

Among all sirtuins, SIRT1 has been the most extensively studied in cardiovascular diseases. SIRT1 is the homolog of *S. cerevisiae* Sir2 and is primarily localized in the nucleus (Luo et al., 2001; Vaziri et al., 2001; Michishita et al., 2005). Studies on adult mouse hearts demonstrated that SIRT1 can be translocated from the nucleus to the cytosol (Tanno et al., 2007). SIRT1 upregulation is associated with cardioprotection since heart-specific SIRT1 overexpression protects the heart against cardiac dysfunction and cell death (Alcendor et al., 2007), cardiac hypertrophy (Planavila et al., 2011), and mediates the cardioprotective effects of ischemic preconditioning (Nadtochiy et al., 2011). One of the mechanisms underlying the cardioprotective effects of SIRT1 is mediated through the nuclear induction of the MnSOD (or SOD2) expression (Tanno et al., 2010).

SIRT6 is localized in the nucleus where it is tightly bound to chromatin (Liszt et al., 2005; Michishita et al., 2005; Mostoslavsky et al., 2006; Sundaresan et al., 2012). In addition to its main role in regulating DNA transcription via histone deacetylation (Michishita et al., 2008, 2009; Yang et al., 2009), SIRT6 also possesses ADP-ribosyltransferase activity (Liszt et al., 2005). SIRT6 has been suggested to be crucial for cell survival as SIRT6 KO mice died within 24 days of birth due to degenerative processes (Mostoslavsky et al., 2006). On the other hand, when SIRT6 KO mice were crossbred, the life-span increased to 1 year, but they spontaneously developed cardiac hypertrophy (Sundaresan et al., 2012). Also, SIRT6 expression was downregulated in the failing human hearts as well as in a mouse model of HF (Sundaresan et al., 2012). Thus, the high mortality and spontaneous development of hypertrophy in SIRT6 deficient animals suggest that SIRT6 has a crucial role in cardiac function.

SIRT7 is primarily localized in the nucleus/nucleolus although it was also detected in the cytoplasm of fibroblasts (Michishita et al., 2005; Kiran et al., 2013). SIRT7 deacetylase and deacylase activity can be regulated by dsDNA (Tong et al., 2016) and RNA (Tong et al., 2017). Interestingly, SIRT7 has been found to regulate mitochondrial biogenesis through the regulation of nuclear-encoded mitochondrial genes. Additionally, SIRT7 deficient mice exhibited a lower mitochondrial oxygen consumption rate in the heart (Ryu et al., 2014) and a 50% decrease in lifespan which correlated with an increase in cardiac hypertrophy, fibrosis, and apoptosis (Vakhrusheva et al., 2008).

### Mitochondrial Sirtuins (SIRT3, SIRT4, and SIRT5)

#### SIRT3

The expression of SIRT3 is high in the heart, and experimental studies demonstrated that mitochondrial protein acetylation only increases in the absence of SIRT3 but not SIRT4 or SIRT5 (Lombard et al., 2007). SIRT3 has been implicated in various cardiac pathologies and it deacetylates multiple enzymes in mitochondrial metabolism (Sol et al., 2012). The heart, along with the brain and skeletal muscle, is the organ most affected by SIRT3 deficiency. SIRT3 KO animals display more than a twofold increase in mitochondrial protein acetylation in these organs, suggesting a critical role for SIRT3 in regulating cardiac mitochondria metabolism (Dittenhafer-Reed et al., 2015). Also, NADH levels are higher in SIRT3 deficient hearts, thus indicating that SIRT3 has a role in the maintenance of mitochondrial redox potential and metabolism (Koentges et al., 2015). However, compared to other tissues such as the liver, the role of SIRT3 in cardiac mitochondria is not well-characterized. Tissue-specific differences in mitochondrial regulation by SIRT3 (Dittenhafer-Reed et al., 2015) further highlight the importance of studies elucidating the role of SIRT3 in the heart. Since SIRT3 is the main sirtuin involved in acetylation/deacetylation of mitochondrial proteins, this review focuses on the contribution of SIRT3 to mitochondrial metabolism and function in the heart.

#### SIRT4

Mitochondrial SIRT4 and SIRT5 resemble prokaryotic sirtuins (Frye, 1999) which indicate a bacterial ancestry of the mitochondria. SIRT4 has no deacetylase activity, but it is an ADP-ribosyltransferase (Ahuja et al., 2007) and is involved in the regulation of lipid metabolism (Nasrin et al., 2010; Laurent et al., 2013). In the heart, SIRT4 mediates the detrimental effects of cardiac hypertrophy including ROS production and fibrosis (Luo et al., 2017). However, hypoxia reduces the expression of SIRT4, and its overexpression protected H9c2 cells against hypoxia-induced cell death (Liu et al., 2013a). It is likely that SIRT4 participates in regulating cardioprotective pathways although the mechanisms by which it exerts the beneficial effects remain unknown.

#### SIRT5

SIRT5 is a weak deacetylase but has strong desuccinylase, demalonylase, and deglutarylase activities (Du et al., 2011; Rardin et al., 2013a; Tan et al., 2014; Boylston et al., 2015). It has also been reported that SIRT5 can function as a deglutarylate (Tan et al., 2014). SIRT5 has been implicated in regulating different aspects of mitochondrial metabolism (Nakagawa et al., 2009; Park et al., 2013; Rardin et al., 2013a; Nishida et al., 2015). In H9c2 cells, the expression of SIRT5 is reduced in response to hydrogen peroxide. Downregulation of SIRT5 reduces cell viability in response to oxidative stress, and SIRT5 overexpression protects these cells (Liu et al., 2013a). In the heart, SIRT5 deficiency renders mice more sensitive to IR injury (Boylston et al., 2015). The roles of SIRT4 and SIRT5 in the heart are discussed elsewhere (Bindu et al., 2016; Bugger et al., 2016).

## Acetylation of Mitochondrial Proteins

Mitochondria metabolism and function is highly regulated by protein acetylation. Pioneering studies have reported that 30% of cytosolic and 15% of mitochondrial proteins are acetylated in the rat heart (Lundby et al., 2012). However, analysis of the mitochondrial acetylome in detail by using advanced technology revealed that over 60% of mitochondrial proteins contain acetylation sites and that most of these proteins are involved in regulating energy metabolism, particularly, fatty acid metabolism, TCA cycle, ETC and OXPHOS (Guan and Xiong, 2011; Foster et al., 2013; Hebert et al., 2013; Baeza et al., 2016) (**Table 2**). Acetylation of mitochondrial proteins occurs through both, enzymatic and non-enzymatic pathways. Enzymatic acetylation involves the GNAT family of acetyltransferases, which include acetyl-CoA acetyltransferase (ACAT1) (Fan et al., 2014) and GCN5L1 (Scott et al., 2012; Thapa et al., 2017). Non-enzymatic protein acetylation relies on the spontaneous transfer of an acetyl group from acetyl-CoA to a lysine residue and therefore, does not rely on the activity of an enzyme (Baeza et al., 2015; Weinert et al., 2015). Acetylation of mitochondrial proteins occurs primarily through the non-enzymatic mechanism due to the high abundance of acetyl-CoA in the matrix (Baeza et al., 2015). Therefore, deacetylation by sirtuins, particularly SIRT3, is the main mechanism regulating protein acetylation in mitochondria.

**Table 2:** Regulation of mitochondrial bioenergetics in the heart by SIRT3.

SIRT3 targets	Effect of deacetylation	Confirmed by *in vitro* deacetylase assays	Biological function	Reference
Acetyl-CoA synthetase 2	↑	Yes	Acetate metabolism	Hallows et al., 2006 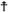 ; Schwer et al., 2006 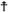
Glutamate dehydrogenase	↑	Yes	Amino acid catabolism	Lombard et al., 2007 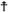 ; Schlicker et al., 2008 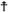
FOXO3a	↑	Yes	Transcription	Sundaresan et al., 2009
LCAD	↑	Yes	Fatty acid metabolism	Hirschey et al., 2010 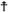 ; Chen et al., 2015; Koentges et al., 2015
PDH	↑	Yes	TCA cycle	Mori et al., 2013; Ozden et al., 2014; Zhang et al., 2018
Aconitase	↓	No	TCA cycle	Fernandes et al., 2015
IDH	↑ ↓ (?)	Yes	TCA cycle; antioxidant system	Schlicker et al., 2008 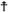 ; Nadtochiy et al., 2017
MDH	N.C.	Yes	TCA Cycle	Nadtochiy et al., 2017 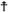
Complex I	↑/N.C. (?)	Yes	ETC	Ahn et al., 2008 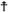 ; Porter et al., 2014; Koentges et al., 2015, 2016; Parodi-Rullán et al., 2017
Complex II	↑	Yes	ETC	Finley et al., 2011 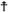 ; Parodi-Rullán et al., 2017
Complex III	N.C. (?)	No	ETC	Koentges et al., 2015; Parodi-Rullán et al., 2017
Complex IV	N.C. (?)	No	ETC	Koentges et al., 2015; Parodi-Rullán et al., 2017
Complex V	↑	No	OXPHOS	Pillai et al., 2015
Oxoguanine glycosylase	↑	No	BER	Pillai et al., 2016, 2017
MnSOD (SOD2)	↑/N.C.	Yes	Mitochondrial antioxidant system	Sundaresan et al., 2009; Tao et al., 2010 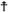 ; Porter et al., 2014; Pillai et al., 2015, 2017; Yang et al., 2016b; Parodi-Rullán et al., 2017
Mitochondrial ribosomal protein L10 (MRPL10)	↓	Yes	Protein synthesis	Yang et al., 2010 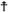
Liver kinase B1 (LKB1)	?	Yes	AMPK Pathway	Pillai et al., 2010
Ku70	?	Yes	DNA repair	Sundaresan et al., 2008

*N.C., no change. cccPurified protein only (not in the heart).*

## SIRT3 Regulates Mitochondrial Bioenergetics

### SIRT3 and Fatty Acid Oxidation

Acyl-CoA dehydrogenases catalyze the first reaction of FAO in the mitochondria. Among all acyl-CoA dehydrogenases, the role of SIRT3 in the regulation of the activity of the LCAD has been extensively studied. *In vitro* studies demonstrated that acetylation of LCAD reduced its activity by 75% and that SIRT3 was able to deacetylate LCAD and increase its activity. Two additional acyl-CoA dehydrogenases (ACADs), medium-chain acyl-CoA dehydrogenases (MCAD), and ACAD9, were found to be SIRT3 substrates, but further studies are necessary to confirm the role of acetylation/deacetylation of these enzymes in FAO activity (Bharathi et al., 2013).

In SIRT3^-/-^ mice, liver mitochondria have 71% of the proteins involved in FAO with increased acetylation (Rardin et al., 2013b). These mitochondria have higher FAO intermediates suggesting decreased levels of FAO. Indeed, only long-chain, but not medium and short chain, fatty acids accumulated in the plasma and in the liver suggesting that acetylation likely plays a role in regulating LCAD activity. Elucidation of LCAD activity in SIRT3 deficient liver mitochondria demonstrated that LCAD is a target of SIRT3 and that acetylation reduced its activity by 53%. Administration of SIRT3 was able to restore LCAD activity (Hirschey et al., 2011). In addition to the liver, several cell types have demonstrated a role of SIRT3 in LCAD regulation (Sol et al., 2012).

Importantly, several groups have demonstrated the role of LCAD acetylation in the heart (Koentges et al., 2015). SIRT3 deficient animals demonstrate 33% lower FAO in the heart (Hirschey et al., 2010) and increased LCAD acetylation (Chen et al., 2015) when compared to WT animals suggesting that cardiac LCAD acetylation could have an inhibitory effect on the enzymatic activity (Koentges et al., 2015). LCAD acetylation has been found in several models of cardiac diseases such as hypertrophy (Chen et al., 2015) and HF (Grillon et al., 2012). Additionally, using expression vectors in H9c2 cells, murine LCAD was able to interact with SIRT3 and overexpression of SIRT3 prevented hypertrophy-characterized lipid accumulation (Chen et al., 2015), further supporting that acetylation has an inhibitory role on LCAD in the heart. In contrast, in the hearts of animals fed a high-fat diet, LCAD was hyperacetylated, and FAO was increased (Abo Alrob and Lopaschuk, 2014).

Conflicting evidence exists on the effect of LCAD acetylation on its enzymatic activity in the heart. These results could represent an additive effect of several PTMs of the enzyme, which could influence its activity differently between pathologies. For example, in cardiac diseases, acetylation could reduce LCAD enzymatic activity due to the combination with another PTM, different from the one observed with a high-fat diet. These data suggest that although acetylation is an important mediator of cardiac FAO metabolism, its effect could vary among pathologies.

### SIRT3 as a Regulator of the TCA Cycle

The liver of SIRT3 KO mice has 43% of the proteins involved in the TCA cycle with increased acetylation (Rardin et al., 2013b). Therefore, it is evident that acetylation could have a profound role in regulating energy metabolism in the TCA cycle. In the next subsections, we will discuss the data available on the regulation of the TCA cycle enzymes by SIRT3. The role of SIRT3 in regulating the SDH activity is discussed in complex II.

#### Pyruvate Dehydrogenase

The PDH complex is composed of three enzymes which catalyze the rate-limiting step of pyruvate decarboxylation (pyruvate to acetyl-CoA) to enter the TCA cycle. This process is critical as it links glycolysis to aerobic energy production through the TCA cycle. Hence, regulation of the enzymatic activity of PDH determines substrate flux through the ETC coupled with OXPHOS in mitochondria.

Several studies have revealed that acetylation decreases PDH activity in the liver, skeletal muscle, and MEFs derived from SIRT3 deficient animals (Sol et al., 2012; Jing et al., 2013). In addition, some of these studies have established that PDH deacetylation is directly regulated by SIRT3 (Jing et al., 2013; Fan et al., 2014). Indeed, SIRT3 deficient animals demonstrated increased accumulation of pyruvate in skeletal muscle mitochondria consistent with inhibition of pyruvate decarboxylation (Jing et al., 2013). Importantly, a physical interaction was observed between SIRT3 and PDH suggesting that its enzymatic activity is regulated by SIRT3 (Ozden et al., 2014). However, only a few studies have focused on the role of SIRT3 in regulating PDH activity in the heart (Alrob et al., 2014). In the heart, increased protein acetylation due to SIRT3 deficiency or angiotensin II treatment was associated with decreased PDH activity (Mori et al., 2013; Ozden et al., 2014; Zhang et al., 2018). Due to the important role in mitochondrial bioenergetics, elucidating the role of SIRT3 in the regulation of PDH activity in the heart needs further studies.

#### Aconitase

Aconitase is an enzyme of TCA cycle that catalyzes the isomerization of citrate to isocitrate. In SIRT3^-/-^ mice-derived MEF, aconitase was significantly hyperacetylated when compared to MEF derived from WT animals (Hebert et al., 2013). In an experimental model of HF and in human HF samples, an increase in aconitase acetylation was observed when compared to sham (Horton et al., 2016). Aconitase hyperacetylation was able to increase its activity in the heart and SIRT3, in the presence of NAD^+^, was able to deacetylate and reduce its activity (Fernandes et al., 2015). Although acetylation is regarded as an inhibitory PTM, evidence suggests that acetylation of aconitase in the heart increases its activity.

#### Isocitrate Dehydrogenase

Isocitrate dehydrogenase catalyzes the formation of α-ketoglutarate (α-KG) from isocitrate by utilizing NADP^+^ and generating NADPH in the TCA cycle. Acetylation of IDH2 was demonstrated in HeLa cells and liver mitochondria isolated from human and mouse tissues (Kim et al., 2006; Zhao et al., 2010; Hebert et al., 2013; Rardin et al., 2013b). SIRT3 deficiency increased IDH2 acetylation in MEF (Hebert et al., 2013) and liver mitochondria (Someya et al., 2010; Hebert et al., 2013; Rardin et al., 2013b). Several studies have reported that deacetylation of IDH2 by SIRT3 increases its activity *in vitro* using purified porcine IDH2 (Schlicker et al., 2008) and in HEK293 cells (Someya et al., 2010; Yu et al., 2012). However, IDH2 activity was only found reduced in the liver but not in the inner ear and brain of SIRT3 KO mice (Someya et al., 2010). In contrast, it was recently reported that *in vitro* acetylation of purified IDH2 from the porcine heart increased its activity (Nadtochiy et al., 2017). These results suggest that acetylation of IDH2 influences its enzymatic activity in a tissue-dependent manner; deacetylation increases its activity in the kidney and liver but decreases its activity in the heart and has no effect in the inner ear and brain.

#### Malate Dehydrogenase

Malate dehydrogenase is one of four dehydrogenases involved in the TCA cycle. It catalyzes the oxidation of malate to oxaloacetate, and the latter re-enters the TCA cycle to produce citrate. Several studies revealed that MDH possesses the ability to be acetylated (Kim et al., 2006; Zhao et al., 2010; Grillon et al., 2012). MDH2 was found hyperacetylated in SIRT3 deficient mouse liver mitochondria (Hebert et al., 2013) and MEF (Sol et al., 2012). A 66-fold increase in acetylation levels at Lys-239 was reported in SIRT3 deficient MEF suggesting that acetylation of this residue might have an important role in regulating its enzymatic activity. Indeed, when the lysine was substituted for glutamine, mimicking the acetylation state, a marked reduction of MDH activity was observed (Hebert et al., 2013). These studies provide evidence that MDH activity is reduced because of its acetylation. However, Zhao et al. (2010) reported that acetylation of MDH increased its enzymatic activity in Chang liver (HeLa derivative) and HEK293 cells. Interestingly, neither acetylation nor deacetylation had any effect on the enzymatic activity of purified bovine heart MDH2 (Nadtochiy et al., 2017). These results suggest that, like IDH2, the effects of acetylation on the MDH enzymatic activity are tissue-dependent. In addition, differences in experimental models of acetylation, i.e., acetyl mimetics, *in vitro* acetylation, or SIRT3 deficiency, could account for variations in results (Nadtochiy et al., 2017).

### Regulation of ETC by SIRT3

#### Complex I

Complex I is composed of 45 subunits including 14 core and 31 supernumerary (accessory) subunits (Zhu et al., 2016). It transfers electrons from NADH to coenzyme Q_10_ expelling 4H^+^ into the intermembrane space thereby contributing to the mitochondrial membrane potential (Ψ_m_). Importantly, complex I is one of the most important contributors to ROS production (Brand, 2010). It is also involved in the pathogenesis of various cardiovascular diseases (Neubauer, 2007) and plays a crucial role in mitochondria-mediated cell death.

In the human cell line HEK293T, treatment with NR, a pyridine-nucleoside form of vitamin B_3_ that functions as a NAD^+^ precursor, decreased the acetylation levels of the complex I subunit NDUFA9 (Cantó et al., 2012), possibly through activation of SIRT3. This subunit was found acetylated in SIRT3 deficient liver mitochondria and MEF (Ahn et al., 2008). Other studies demonstrated a direct interaction between SIRT3 and two subunits of complex I, NDUFA11 and NDUFS8 (Finley et al., 2011). Furthermore, complex I acetylation decreased its activity by 20% in SIRT3 deficient liver mitochondria. In HeLa cells, treatment with NAM (SIRT inhibitor) increased complex I acetylation, and the immunocaptured complex I was deacetylated *in vitro* in the presence of exogenous SIRT3 but not SIRT4 (Ahn et al., 2008). In contrast, other studies found that skeletal muscle or liver-specific SIRT3 deletion did not affect complex I activity (Fernandez-Marcos et al., 2012). Altogether, these results suggest that SIRT3 can interact and regulate complex I activity in various non-cardiac tissues.

In the heart, cardiac-specific deletion of NDUFS4 reduced complex I activity and mitochondrial respiration by 75% and 45%, respectively. The reduced complex I activity was associated with a 50% decrease in the NAD^+^/NADH ratio leading to inhibition of SIRT3 activity. Mitochondrial proteins from these hearts, including NDUFA9, were found to be hyperacetylated. Conversely, overexpression of SIRT3 was able to reduce protein acetylation (Karamanlidis et al., 2013). In contrast, SIRT3^-/-^ hearts have decreased mitochondrial respiration for complex I (Koentges et al., 2015) and increased levels of NADH (Nadtochiy et al., 2017). These results suggest a strong interdependence of OXPHOS and protein acetylation for the maintenance of mitochondrial function.

Downregulation of SIRT3 increases the sensitivity of cardiac cells to oxidative and energy stress. IR significantly reduced complex I activity in H9c2 cardiomyoblast cells with SIRT3 knockdown (Porter et al., 2014). Likewise, *ex vivo* IR decreased complex I activity (Porter et al., 2014) and state 3 respiration (Koentges et al., 2016) in SIRT3 deficient hearts. We found no difference in complex I activity between SIRT3^-/-^ and WT hearts subjected to IR (Parodi-Rullán et al., 2017). Likewise, no differences were observed in complex I activity after 7 days of permanent coronary artery ligation (Koentges et al., 2016). Lastly, several complex I subunits were found to be hyperacetylated in an experimental model of HF and in the failing human heart (Horton et al., 2016). Altogether, these studies suggest that complex I acetylation is present during cardiac pathologies thereby portraying SIRT3 as a key player.

#### Complex II

Succinate dehydrogenase or complex II is a key enzyme of the TCA cycle and has two main roles in the mitochondria: (i) catalyzes the oxidation of succinate to fumarate, and (ii) reduces ubiquinone to ubiquinol. It is comprised of four subunits: two catalytic subunits, SDHA and SDHB, attached to the IMM and facing into the matrix, and two membrane-anchored subunits, SDHC and SDHD. The SDHA subunit is mostly involved in the oxidation of succinate to fumarate, while the other three subunits catalyze the reduction of ubiquinone to ubiquinol.

In liver mitochondria, SIRT3 deficiency increased SDHA acetylation (Rardin et al., 2013b) and SIRT3 was able to deacetylate SDHA, but it did not affect its activity (Finley et al., 2011). On the other hand, there was no difference in acetylation in immunocaptured complex II from WT and SIRT3 deficient livers (Ahn et al., 2008). Also, these studies found no interaction between complex II and SIRT3, and SIRT3 deletion did not affect complex II-mediated respiration. In contrast, other groups reported that SDHA hyperacetylation decreased its electron transferring activity (Cimen et al., 2010; Finley et al., 2011). Additionally, SDHA was shown to be a target for SIRT3 in SIRT3 KO embryonic fibroblasts (Sol et al., 2012). In the absence of SIRT3, SDH activity was reduced by 25% (Finley et al., 2011) and succinate accumulation increased (Hebert et al., 2013) suggesting inhibition of the SDHA subunit.

Similarly, SIRT3 deficient hearts demonstrated an increase in SDH acetylation (Koentges et al., 2015). Moreover, the complex II activity in SIRT3 KO hearts was lower than WT hearts after *ex vivo* IR, possibly due to complex II hyperacetylation (Parodi-Rullán et al., 2017). In the failing human heart, as well as in mice with HF, SDHA acetylation correlated with decreased complex II-mediated respiration and SDH activity (Horton et al., 2016). Thus, acetylation of complex II, like complex I, is associated with a reduced activity in the heart due to a possible downregulation of SIRT3.

#### Complexes III and IV

Among ETC complexes, acetylation of complexes III and IV and their possible regulation by SIRT3 has been less studied. Cytochrome c reductase or complex III is composed of 11 subunits and contains two monomeric units. Cytochrome c oxidase or complex IV is the final electron acceptor of the ETC; it transfers electrons from cytochrome c to oxygen, thereby producing water at the expense of oxygen.

Skeletal muscle- or liver-specific SIRT3 deletion did not affect complex IV activity (Fernandez-Marcos et al., 2012). Furthermore, 3-day differentiated myoblasts with SIRT3 ablation displayed a decrease in the complex IV activity (Abdel Khalek et al., 2014). In the heart, subunits of complex III and complex IV were differentially acetylated in SIRT3 KO mice (Koentges et al., 2015). The cytochrome c oxidase subunit 4 isoform 1 was found acetylated in an animal model of HF (Horton et al., 2016). Interestingly, several subunits of complexes III and IV were found to be hyperacetylated in the failing human heart (Horton et al., 2016). However, there was no difference between the enzymatic activity of complexes III and IV of SIRT3^-/-^ and WT hearts exposed to IR (Parodi-Rullán et al., 2017). Although existing studies demonstrate acetylation of complexes III and IV, further studies are required to establish the effect of acetylation on the activity of these complexes in the heart.

#### Complex V

The main function of the F_1_F_O_-ATP synthase or complex V is to synthesize ATP from ADP and P_i_ through the process known as OXPHOS. Indeed, several studies have demonstrated the potential role of SIRT3 in regulating ATP production. In HEK293T and human osteosarcoma cells, SIRT3 downregulation increased the acetylation of the OSCP and alpha subunits of complex V, and this was associated with decreased activity and ATP levels (Wu et al., 2013). Several groups have reported that SIRT3 physically interacted with the OSCP subunit in human osteosarcoma cells (Wu et al., 2013), HEK293T cells (Wu et al., 2013; Yang et al., 2016a), and HCT116 cells (Vassilopoulos et al., 2014). Furthermore, recent studies suggested that the OSCP-SIRT3 interaction is amongst the most specific SIRT3 interactions (Yang et al., 2016a). Also, there is evidence that SIRT3 interacts with other subunits of the ATP synthase (Finley et al., 2011; Vassilopoulos et al., 2014). SIRT3 deficient MEF produced 30% less ATP and expression of SIRT3 restored the ATP levels (Ahn et al., 2008). In contrast, skeletal muscle or liver-specific SIRT3 deletion did not affect the levels of ATP (Fernandez-Marcos et al., 2012). This data demonstrates that SIRT3 not only interacts and deacetylates various subunits of complex V, but it can also regulate ATP production.

Recent studies suggest that SIRT3 is involved in the regulation of ATP production in the heart through the deacetylation of mitochondrial proteins. ATP levels were reduced in the heart of SIRT3 deficient mice (Ahn et al., 2008; Koentges et al., 2016) and the effect remained unchanged after *ex vivo* IR (Koentges et al., 2015). HKL, a SIRT3 activator, reduced OSCP acetylation in primary rat cardiomyocytes (Pillai et al., 2015) suggesting that the OSCP subunit is also a SIRT3 target in cardiac cells. Most importantly, in animal models of HF and in the failing human heart, several subunits of the F_1_F_O_-ATP synthase displayed an increase in acetylation (Grillon et al., 2012; Horton et al., 2016). Reduced ATP production is one of the hallmarks of HF (Long et al., 2015); therefore, these findings provide strong evidence that increased F_1_F_O_-ATP synthase acetylation due to reduced SIRT3 activity could be a key mediator of mitochondria-mediated cardiac dysfunction.

## The Role of SIRT3 in the Regulation of Mitochondrial ROS and Redox State

Overexpression of SIRT3 has been demonstrated to prevent ROS accumulation in cardiomyocytes in response to different stressors such as the anticancer agent triptolide (TP) (Yang et al., 2016b) and the α_1_-adrenergic receptor agonist phenylephrine (Sundaresan et al., 2009). Indeed, SIRT3 deficient cardiomyocytes produced double the levels of ROS than WT cells with and without phenylephrine stimulation (Sundaresan et al., 2009). In neonatal rat cardiomyocytes, phenylephrine-induced ROS stimulation was abolished by treatment with the sirtuin activator NAD^+^ (Pillai et al., 2010). Although the mitochondrial ROS levels were no different between SIRT3^-/-^ and WT hearts (Koentges et al., 2015; Parodi-Rullán et al., 2017), IR induced an increase in ROS levels in SIRT3 deficient mitochondria (Parodi-Rullán et al., 2017). Resveratrol, a potent SIRT1 activator, prevented H_2_O_2_-induced cell death but this effect was absent in the presence of NAM (Chen et al., 2013). Nevertheless, these studies suggest that SIRT3 can regulate mitochondrial ROS levels, at least during cardiac stress, through the regulation of the mitochondrial antioxidant system.

Mitochondrial DNA is susceptible to oxidative damage when the ROS levels exceed the antioxidant capacity of mitochondria. Oxidative damage to DNA results in the oxidation of guanine (G) to 8-oxo-deoxyguanosine (8-oxo-dG) which leads to an abnormal G:T transversion eventually creating a mutation. This type of lesion is repaired by the BER pathway which is also present in the mitochondria (Alexeyev et al., 2013), and oxoguanine glycosylase and apurinic/apyrimidinic endonuclease 1 are the enzymes involved in the BER pathway. Although the BER pathway is not directly involved in ROS reduction, it repairs ROS-induced mtDNA lesions. SIRT3 has been shown to regulate the activity of enzymes involved in BER.

In rat neonatal cardiomyocytes, the anticancer drug doxorubicin increased mtDNA lesions and oxoguanine glycosylase acetylation. Activation of SIRT3 by HKL reduced 8-oxo-dG lesions in cardiomyocytes (Pillai et al., 2017). SIRT3 can interact with oxoguanine glycosylase. In the absence of SIRT3, doxorubicin increased the levels of 8-oxo-dG, which was abrogated by SIRT3 overexpression. Cardiac-specific SIRT3 overexpression increased the resistance of cardiomyocytes to doxorubicin-induced mtDNA damage (Pillai et al., 2017), and conversely, the hearts of SIRT3 deficient mice were more susceptible to doxorubicin-induced mtDNA damage (Pillai et al., 2016). Thus, SIRT3 is involved in regulating the mitochondrial antioxidant system and the BER pathway to prevent ROS-induced protein oxidation and mtDNA damage. In response to energy and oxidative stress, ROS production likely exceeds the antioxidant capacity of the mitochondria due to acetylation-induced inactivation of mitochondrial antioxidant enzymes.

### Mitochondrial Antioxidant Enzymes

A growing number of studies suggest a possible link between SIRT3 and the mitochondrial antioxidant system. SIRT3 deficiency increased superoxide levels in rat myoblastic cells (Abdel Khalek et al., 2014), MEF, and liver tissue (Tao et al., 2010). The increased ROS was associated with low activity of MnSOD (Tao et al., 2010; Abdel Khalek et al., 2014) and its hyperacetylation (Tao et al., 2010). SIRT3 expression was able to deacetylate MnSOD and increase its activity in SIRT3^-/-^ MEF (Tao et al., 2010) and reduce mitochondrial and cellular ROS (Qiu et al., 2010; Tao et al., 2010). Similar results were also found in HEK293T (Qiu et al., 2010; Cantó et al., 2012) and BREC cells (Gao et al., 2016). These results suggest that MnSOD is a direct target of SIRT3. Acetylation of MnSOD renders the enzyme inactive whereas deacetylation by SIRT3 activates it, therefore decreasing superoxide levels. Although confounding evidence suggests that SIRT3 regulates MnSOD, it should be noted that tissue-specific deletion of SIRT3 in the muscle or liver did not result in any difference between mitochondrial MnSOD and catalase activity (Fernandez-Marcos et al., 2012).

Studies on the heart also suggested a potential role of SIRT3 in regulating mitochondrial ROS. Under physiological conditions, SIRT3 overexpression increased MnSOD but not catalase activity in H9c2 cardiomyoblastic cells (Yang et al., 2016b). MnSOD activity was reduced in the heart of SIRT3^-/+^ mice (Porter et al., 2014) although other studies were unable to detect a difference in MnSOD activity between SIRT3^-/-^ and WT hearts (Parodi-Rullán et al., 2017). Interestingly, these studies revealed low MnSOD activity in SIRT3^-/+^ (Porter et al., 2014) and SIRT3^-/-^ (Parodi-Rullán et al., 2017) hearts after oxidative stress induced by *ex vivo* IR. The reduced MnSOD activity was associated with increased oxidative damage to mitochondrial proteins (Parodi-Rullán et al., 2017). Conversely, treatment with NAD^+^, a sirtuin activator, recovered MnSOD activity in WT hearts after IR (Zhang et al., 2016).

In rat neonatal cardiomyocytes, HKL reduced H_2_O_2_-induced MnSOD acetylation which was associated with decreased ROS levels and cell death. These effects of HKL were absent in SIRT3 silenced neonatal cardiomyocytes (Pillai et al., 2015). Interestingly, HKL treatment or SIRT3 overexpression protected neonatal cardiomyocytes against doxorubicin-induced damage through reduction of ROS levels (Pillai et al., 2016, 2017). Likewise, isoproterenol and angiotensin II diminished both, expression and activity of MnSOD and catalase in cardiomyocytes. However, these effects were not observed in SIRT3 overexpressed cells (Sundaresan et al., 2009). SIRT3 overexpression also prevented the decrease in catalase and MnSOD activity in response to TP-induced stress (Yang et al., 2016b). These studies demonstrate a strong link between SIRT3 activity and MnSOD acetylation; SIRT3 deacetylates and activates MnSOD thereby reducing mitochondrial ROS levels.

### Mitochondrial Redox State

**FIGURE 1:**
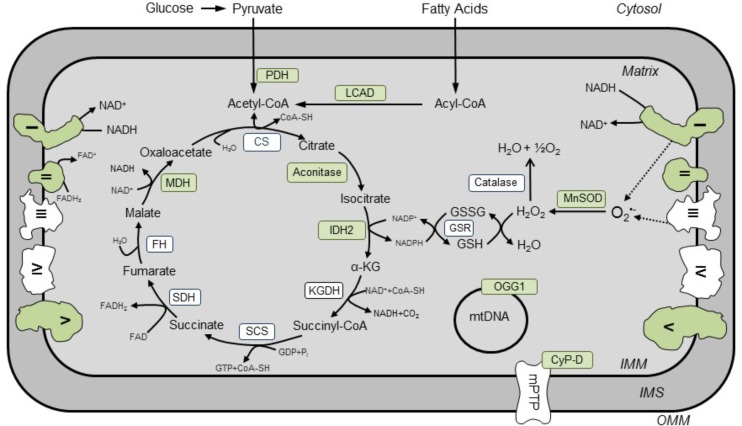
Known SIRT3 targets in cardiac mitochondria. Enzyme-targets of SIRT3 in the heart are shown in green. Activities of enzymes (*shown in green*) involved in the TCA cycle (aconitase, MDH, IDH2), ETC (complexes I and II), OXPHOS (complex V), FAO (LCAD), base excision repair system (OGG1), antioxidant enzymes (MnSOD), mPTP induction (CyP-D), and pyruvate oxidation (PDH) are regulated by SIRT3. CS, citrate synthase; CyP-D, cyclophilin D; FH, fumarate hydratase; GSR, NADPH-dependent glutathione reductase; IDH2, isocitrate dehydrogenase; KGDH, α-ketoglutarate dehydrogenase; LCAD, long-chain acyl-CoA dehydrogenase; MDH, malate dehydrogenase, MnSOD, Mg^2+^-dependent mitochondrial superoxide dismutase; OGG1, oxoguanine glycosylase; PDH, pyruvate dehydrogenase; SCS, succinyl CoA synthetase; SDH, succinate dehydrogenase.

Although IDH is a TCA cycle enzyme, it also serves as a mitochondrial source of NADPH and thus, is the main regulator of the NADPH-dependent glutathione reductase (GSR). GSR is involved in regenerating glutathione (GSH) from glutathione disulfide (GSSG), and therefore in H_2_O_2_ detoxification with glutathione peroxidase (**Figure 1**). Thus, high levels of IDH2 increase the NADPH levels and consequently, the GSH pool, augmenting the mitochondrial detoxification system. Indeed, IDH2 contributes 25% of the total mitochondrial NADPH (Yu et al., 2012). As discussed previously (see section “Isocitrate Dehydrogenase”), IDH2 is a target of SIRT3, and its deacetylation increased NADPH levels in HEK293 cells (Someya et al., 2010; Yu et al., 2012). This increase in NADPH levels protected the cells against H_2_O_2_-induced oxidative damage (Yu et al., 2012). Interestingly, although CR increased the GSH to GSSH ratio in the WT mouse liver, brain, and inner ear, this effect was not observed in SIRT3 deficient tissues (Someya et al., 2010). Thus, a growing body of studies provides evidence in favor of indirect actions of SIRT3 in regulating the glutathione system through IDH as a source of NADPH. However, the precise role of SIRT3 in the glutathione system in the heart remains to be elucidated.

### SIRT3 and Caloric Restriction in the Heart

Caloric restriction, or a reduction of caloric intake of 30% or more without malnutrition, has been linked to lifespan extension. It has been shown to induce changes in mitochondrial protein acetylation (Schwer et al., 2009; Shinmura et al., 2011). However, the heart, along with the kidney and brain, demonstrates only subtle changes in mitochondrial protein acetylation after CR in comparison to the liver and brown adipose tissue. Interestingly, changes in acetylation in response to CR vary among tissues, where, in contrast to the liver, CR reduced mitochondrial protein acetylation in the brown adipose tissue (Schwer et al., 2009). Although the heart, skeletal muscle, and brain contain fewer acetylation sites in mitochondrial proteins in comparison to the liver and kidney, these sites are more dynamic in response to 48 h of fasting suggesting an essential role of CR-mediated acetylation in mitochondrial metabolism (Dittenhafer-Reed et al., 2015).

Since SIRT3 is a NAD^+^-dependent deacetylase, the increase in mitochondrial acetylation observed with aging is probably due to low NAD^+^ levels and/or reduced SIRT3 expression. However, CR increased SIRT3 expression in liver (Hirschey et al., 2010; Someya et al., 2010; Hallows et al., 2011; Hebert et al., 2013), skeletal muscle (Palacios et al., 2009), and in white (Shi et al., 2005) and brown adipose tissue (Shi et al., 2005; Hirschey et al., 2010) but not in the heart, kidney or brain (Hirschey et al., 2010). Therefore, it would be equally important to measure, in addition to SIRT3 expression, NAD^+^ availability for SIRT3 activity during CR.

It has been reported that aging decreased the activity of NAM phosphoribosyltransferase (NAMPT), the rate-limiting enzyme for NAD^+^ synthesis, and NAD^+^ levels in the mitochondria (Yoshino et al., 2011) suggesting that the anti-aging effects observed with CR could also be mediated through increasing mitochondrial NAD^+^ levels. Interestingly, rats that underwent fasting for 48 h displayed enhanced mitochondrial NAMPT expression associated with increased NAD^+^ levels in liver mitochondria (Yang et al., 2007). Most importantly, CR was able to increase SIRT3 activity in cardiac mitochondria (Shinmura et al., 2011). Although SIRT3 is essential in the prevention of age-related hearing loss (Someya et al., 2010; Yu et al., 2012; Han and Someya, 2013), there is no data on the role of SIRT3 in mediating the cardioprotective effects of CR. Studies in humans suggest that SIRT3 can be involved in the mediation of lifespan extension (Rose et al., 2003; Bellizzi et al., 2005). Since cardiovascular diseases, particularly HF, are age-associated diseases, it is important to study the potential mechanism by which SIRT3 can mediate the cardiac-related effects of CR on lifespan.

## Regulation of Mitochondrial Dynamics by SIRT3

### Mitochondrial Biogenesis

Mitochondrial biogenesis is a mechanism of mitochondrial turnover, primarily driven by the expression of nuclear-encoded proteins such as the peroxisome PGC-1α. PGC-1α is a member of the nuclear receptor superfamily that mediates the assembly of transcriptional machinery directed to specific DNA sequences. PGC-1α is highly expressed in the skeletal muscle and in the heart. It regulates, through the mitochondrial transcriptional network, expression of genes encoding mitochondrial proteins that are involved in FAO, the TCA cycle, and OXPHOS (*reviewed in* Fan and Evans, 2015). It also regulates the nuclear respiratory factor 1 and 2 (NRF1 and NRF2) which interact with mitochondrial transcription factor A (TFAM). The latter regulates the expression of mtDNA-encoded genes by binding to the light and heavy strand mtDNA promoters (Taherzadeh-Fard et al., 2011). The transcriptional network regulating mitochondrial biogenesis is reduced in cardiac diseases including MI (IR) and HF. Expression of PGC-1α and its downstream factors were diminished in animal models of HF induced by aortic banding (Garnier et al., 2003) and post-MI (Javadov et al., 2006) as well as in patients with HF (Finck and Kelly, 2007; Mistry and Cresci, 2010).

Several studies demonstrated crosstalk between mitochondrial biogenesis and SIRT3 in cardiac pathologies, particularly in cardiac remodeling induced by a hypertrophic response. PGC-1α has been shown to regulate the expression of oxidative stress protective genes through the interaction with the transcriptional activator protein FOXO3a (Olmos et al., 2009), which triggers apoptosis. Interestingly, FOXO3a binding sites have been found in the PGC-1α promoter suggesting that FOXO3a can regulate the expression of PGC-1α (Borniquel et al., 2010). Studies on SIRT3 deficient mice revealed that SIRT3 plays a cardioprotective role in cardiac hypertrophy by suppressing ROS production through the induction of FOXO3a (Sundaresan et al., 2009). Interestingly, oxidative stress *in vitro* did not increase acetylation of PGC-1α in H9c2 cardioblastic cells (Barreto-Torres et al., 2015). However, PGC-1α deficiency resulted in a reduction of SIRT3 gene expression in mice muscle and liver cells. The same study showed that SIRT3 plays an important role in the regulation of ROS detoxifying enzymes such as MnSOD and glutathione peroxidase, which are also associated with PGC-1α (Kong et al., 2010). Studies on human umbilical vein endothelial cells revealed that SIRT3 mediates the deacetylation of FOXO3, inducing its translocation to the nucleus where it upregulates key proteins for mitochondrial biogenesis and quality control such as PGC-1α, mitochondrial transcription factor A, Drp1, Fis1, and Mfn2 (Tseng et al., 2013). The activation of SIRT3 by HKL treatment increased mRNA levels of PGC-1α, reduced ROS production, and improved mitochondrial respiration in a mouse model of pressure overload hypertrophy (Pillai et al., 2015). These findings demonstrate a central role of SIRT3 in regulating mitochondrial biogenesis, reducing oxidative stress and protecting mitochondrial metabolism. Additionally, in SIRT3 deficient mice, inhibition of the interaction between SIRT3, FOXO3a, and Parkin caused a reduction in mitophagy leading to the development of diabetic cardiomyopathy (Yu et al., 2017).

### Mitochondrial Fusion–Fission

Mitochondrial dynamics plays an important role in regulating mitochondrial quality control and it consists of two main processes: (i) fusion: the combination of two mitochondria to maintain their functional and structural integrity through content exchange such as mtDNA, matrix proteins, and essential phospholipids; and (ii) fission: mitochondrial division into two smaller daughter organelles, sometimes referred to as fragmentation.

Mitofusins 1 (Mfn1) and 2 are proteins localized in the OMM that participate in the fusion process (the merging of two individual mitochondria) (Schrepfer and Scorrano, 2016). Studies on Mfn1 and Mfn2 double KO mice demonstrated that the heart became dilated by 5 weeks and developed HF by 7–8 weeks (Chen et al., 2011). The OPA1 protein, which is embedded in the IMM, enables fusion of the IMM through the formation of trans-complexes. In addition to fusion, OPA1 is involved in remodeling and maintaining the cristae structure in the IMM (Rahn et al., 2013). Altogether, fusion proteins play a vital role in maintaining cardiac and contractile functions (Chen et al., 2011) as well as in late cardiac development (Chen et al., 2012).

Mitochondrial fission is mediated by the GTPase protein Drp1 and the adapter protein Fis1 on the OMM. Drp1 is a cytosolic GTPase that is recruited to the OMM during fission. Drp1 homo-oligomerizes to form a constraining chain around the mitochondrion to divide it into two daughter organelles. Mitochondrial fission factor, an OMM protein, functions as a Drp1 receptor that regulates the mitochondrial fission process (van der Bliek et al., 2013).

Alterations in mitochondrial fusion-fission processes are involved in the pathogenesis of cardiac diseases, particularly, MI and HF. Both *in vitro* and *in vivo* models of cardiac hypertrophy and HF decreased OPA1 and Mfn2 expression and increased mitochondrial fragmentation (Fang et al., 2007; Chen et al., 2009). Reduced expression of fusion proteins and increased mitochondrial fission in response to post-infarction remodeling was associated with mitochondrial dysfunction in rats (Javadov et al., 2011). Recent studies using a double KO of Mfn1 and Mfn2 and triple KO of Mfn1, Mfn2, and Drp1 mouse model demonstrated that the complete ablation of mitochondrial dynamics results in cardiac hypertrophy and HF due to diminished mitophagy. Also, alterations in mitochondrial dynamics accelerated mitochondrial senescence (Song et al., 2017).

### SIRT3 and Mitochondrial Dynamics

Several studies have elucidated the role of protein acetylation in mitochondrial dynamics. In human fibroblasts, treatment with NAM enhanced mitochondrial fragmentation and increased the expression of Drp1, Fis1, and Mfn1, though acetylation of these proteins and the mechanisms of their upregulation were not examined (Kang and Hwang, 2009). OPA1 has been shown to contain acetylation sites, and SIRT3 can target OPA1 and regulate its activity. Hyperacetylation of OPA1 inhibits its GTPase activity, while SIRT3 prevents OPA1 acetylation and preserves its activity. In favor of this, electron microscopy imaging of SIRT3 deficient cardiac mitochondria displayed a continuous (fused) OMM between two mitochondria, but weakly bound (not fused) IMM, indicating dysfunctional fusion dynamics (Samant et al., 2014).

Tert-butyl hydroperoxide (t-BHP), an organic peroxide, decreased SIRT3 expression associated with OPA1 acetylation in H9c2 cardiomyoblasts. The acetylation of OPA1 induced its cleavage, inevitably provoking apoptosis (Signorile et al., 2017). Similarly, doxorubicin reduced SIRT3 expression, which was associated with a decrease in OPA1 and Mfn1 expression in mice. Treatment with SIRT3 activator HKL prevented doxorubicin-induced myocardial injury and preserved the expression levels of SIRT3, OPA1, and Mfn1. These studies indicate the importance of maintaining SIRT3 functional activity to prevent the downregulation of mitochondrial fusion proteins (Pillai et al., 2017).

### Mitophagy and SIRT3

Mitophagy is the process of removing damaged, dysfunctional, or abnormal mitochondria through autophagy to maintain proper mitochondrial function in the cell. Under physiological conditions, a membrane potential-dependent protein known as presenilin-associated rhomboid-like protein (PARL) induces cleavage of the mitochondria-localized serine/threonine-protein kinase PTEN-induced putative kinase 1 (PINK1) (Jin and Youle, 2012; Shiau et al., 2017). Under stress, the ability of PARL to cleave PINK1 decreases due to diminished mitochondrial membrane potential (Okatsu et al., 2012). Recent studies also suggested a role of ATP depletion in decreased PINK1 cleavage (Shi and McQuibban, 2017). As a result, uncleaved, full-length PINK1 recruits Parkin, an E3 ubiquitin ligase (Okatsu et al., 2012) which regulates ubiquitination of target proteins such as Nip3-like protein X (NIX). In mammalian cells, the ubiquitination of the latter has been shown to serve as a receptor for mitophagy (Ashrafi and Schwarz, 2013). Other proteins such as Bcl2/adenovirus EB interacting protein 3 (BNIP3), cardiolipin, and FUN14 domain-containing protein-1 (FUNDC1) participate in mitophagy by binding to LC3 (Saito and Sadoshima, 2015).

One of the first indicators of the mitophagy-sirtuin interplay came from NAM-treated primary human fibroblasts. The study found that the sirtuin inhibitor, NAM, induced a decrease in mitochondrial mass and an increase in membrane potential by activating mitophagy to eliminate damaged mitochondria (Kang and Hwang, 2009). Furthermore, the effects of NAM on the mitochondrial quality could be mediated through an increase in the [NAD^+^]/[NADH] ratio leading to SIRT1 activation and induction of mitophagy (Jang et al., 2012). However, mitochondrial sirtuins, particularly SIRT3, were not assessed in these studies. Studies with SIRT3 deficient and overexpressing mice and primary neonatal mouse cardiomyocytes provided evidence that SIRT3 plays a regulatory role in autophagy and mitophagy through deacetylation of FOXO3a and Parkin (Yu et al., 2017). This conclusion was further supported by other studies elucidating the effects of angiotensin II treatment in WT and SIRT3^-/-^ mice where SIRT3 deficiency enhanced the microvascular rarefication (thinning) provoked by angiotensin II (Wei et al., 2017). The study concluded that SIRT3 can stimulate mitophagy by the deacetylation of PINK1 and Parkin and thus, inhibiting angiogenesis and endothelial dysfunction. Nevertheless, the role of SIRT3 in mitophagy remains elusive.

## SIRT3 in Cardiovascular Diseases

### Cardiac Hypertrophy

Cardiac hypertrophy involves the enlargement of the myocardium and development of fibrosis. Animal models of cardiac hypertrophy are induced by surgery and pro-hypertrophic agents that increase pressure and volume overload (stress). Post-infarction remodeling, transverse aortic constriction as well as pro-hypertrophic agents such as angiotensin II, phenylephrine, endothelin-1, and isoproterenol are used to develop animal models of cardiac hypertrophy (Frey and Olson, 2003). Increased ROS production and energy deficiency play a crucial role in cardiac remodeling associated with cardiac dysfunction (Sebastiani et al., 2007). During the progression of cardiac hypertrophy to HF, Akt signaling is linked to alterations in mitochondrial metabolism leading to mitochondrial dysfunction. This pathway is activated through suppression of metabolic transcription regulators such as FOXO and PGC-1α (Wende et al., 2015). Mitochondrial dysfunction reduces metabolic flux thereby reducing NAD^+^ availability, inactivating SIRT3, and consequently provoking an increase in acetylation of proteins such as FOXO3a (Jacobs et al., 2008). Acetylation enhances the susceptibility of FOXO3a to phosphorylation by Akt, thus activating pathways that lead to mitophagy, autophagy, and cell death (Hagenbuchner and Ausserlechner, 2013; Wang et al., 2017). This pathway is involved in the development of the cardiac hypertrophic response which is attenuated by SIRT3 overexpression in mice (Sundaresan et al., 2009). In addition, the AMPK/SIRT1 pathway has been shown to mediate hypertrophy-induced mitochondrial dysfunction in H9c2 cardioblast cells (Hernández et al., 2014) and in the myocardium of spontaneously hypertensive rat (Tang et al., 2014).

The role of mitochondrial protein acetylation in cardiac hypertrophy was confirmed in *in vitro* studies where activation or overexpression of SIRT3 prevented the development of cardiac hypertrophy. Overexpression of SIRT3 was able to prevent cardiac hypertrophy in response to phenylephrine or angiotensin II treatment in H9c2 cardiomyoblast cells (Chen et al., 2015) and rat neonatal cardiomyocytes (Sundaresan et al., 2009). Likewise, pharmacological activation of SIRT3 by treatment with NAD^+^ or HKL prevented phenylephrine- and angiotensin II-induced increase in cell size and attenuated induction of hypertrophic markers in rat neonatal cardiomyocytes (Pillai et al., 2010, 2015). Furthermore, HKL was found to translocate to the mitochondria where it enhanced the activity and expression of SIRT3, therefore reducing acetylation of mitochondrial proteins (Pillai et al., 2015). These findings suggest that HKL exerts anti-hypertrophic effects in a SIRT3-dependent manner.

*In vivo* studies further confirmed the anti-hypertrophic role of SIRT3 in the heart. In a mouse model of angiotensin II-induced cardiac hypertrophy, NAD^+^ prevented the development of hypertrophy and induction of hypertrophic markers (Pillai et al., 2010). Similarly, HKL treatment prevented and also reverted the development of hypertrophy and fibrosis, and the induction of hypertrophic markers (Pillai et al., 2015, 2017), thus indicating that SIRT3 not only protects against cardiac hypertrophy but can also revert it.

SIRT3 deficient hearts develop hypertrophy (Sundaresan et al., 2009; Chen et al., 2015) and cardiac fibrosis (Chen et al., 2015). However, contradicting evidence exists as to whether these animals develop cardiac dysfunction (Sundaresan et al., 2009; Chen et al., 2015). On and all, when these animals were infused with angiotensin II, isoproterenol, or phenylephrine, the hearts developed twice as much cardiac hypertrophy and cardiac dysfunction as their WT counterparts (Sundaresan et al., 2009). In response to transverse aortic constriction, SIRT3 KO mice developed a more severe cardiac hypertrophy accompanied by impaired cardiac function and fibrosis than WT animals (Hafner et al., 2010; Chen et al., 2015; Koentges et al., 2015). Importantly, the cardioprotective effects of HKL against cardiac hypertrophy were abolished in the absence of SIRT3 (Pillai et al., 2015). Also, cardiac-specific SIRT3 overexpression prevented angiotensin II- and doxorubicin-induced cardiac hypertrophy, fibrosis, and fetal gene expression (Sundaresan et al., 2009; Pillai et al., 2016) confirming that SIRT3 has a role in protecting the heart against hypertrophic stimuli.

In conclusion, SIRT3 has a crucial role in the development and progression of cardiac hypertrophy. The pleiotropic effects of SIRT3 activation in the prevention of mitochondrial ROS could be a crucial pathway to the inhibition of cardiac hypertrophy (Sundaresan et al., 2009). However, in addition to ROS, other SIRT3-mediated mechanisms could be involved in preserving mitochondrial and cardiac function in response to hypertrophic stimuli.

### Myocardial Infarction and Ischemia-Reperfusion Injury

Myocardial infarction, or ischemia, is the most common type of cardiovascular disease. Standard treatment after MI is to restore the blood flow to the previously ischemic area, also known as reperfusion. Although restoration of blood flow to the ischemic area is crucial, reperfusion contributes up to 50% of additional damage to the final infarct size; a process also termed as IR injury.

Emerging evidence implicating SIRT3 in a variety of cardioprotective mechanisms led to the postulation that SIRT3 could be implicated in IR injury. H9c2 cells silenced for SIRT3 expression were more susceptible to IR as evidenced by the enhanced release of lactate dehydrogenase, a marker of cellular damage (Porter et al., 2014). Indeed, the absence of one or both SIRT3 alleles in the heart (SIRT3^-/+^ or SIRT3^-/-^) led to a marked susceptibility to *ex vivo* IR. These hearts demonstrated reduced cardiac recovery, and increased infarct size and cell damage (Parodi-Rullán et al., 2017; Porter et al., 2014) after IR. Furthermore, intravenous administration of NAD^+^ before ischemia protected the rat heart from IR injury. These animals demonstrated a significant reduction in infarct size, cardiac troponin levels, and apoptotic markers suggesting the potential role of sirtuins, such as SIRT3, in protecting the heart against IR (Zhang et al., 2016). However, the causative link between SIRT3 and cardioprotection remains to be elucidated. In contrast, another group found no difference between SIRT3^-/-^ and WT hearts in the susceptibility to IR (Koentges et al., 2016). These discrepancies between studies could be explained by differences in heart preparation and duration of ischemia and reperfusion periods. Indeed, the same study demonstrated that *in vivo* coronary artery ligation in the absence of SIRT3 did not render the hearts more prone to cardiac dysfunction than their WT counterparts at 7 days post-MI (Koentges et al., 2016). Nevertheless, the mechanisms mediating cardiac dysfunction are completely different in reperfused and non-reperfused ischemic hearts. SIRT3 activity is affected differently depending on the severity of both, ischemia and reperfusion injury in the heart. Furthermore, pathological mPTP induction can play a central role in mediating the adverse effects of protein acetylation.

### SIRT3 and the mPTP

The mPTP is a non-selective pore that forms in the mitochondria and allows the non-selective passage of ions, solutes, and water up to 1.5 kDa into the mitochondrial matrix. As a result, the increase in IMM permeability induces mitochondrial swelling and rupture of the OMM leading to cell death. The mPTP opening is induced by high matrix Ca^2+^, ROS, and loss of mitochondrial membrane potential; all of these conditions occur at reperfusion (*reviewed in* Javadov et al., 2009; Bernardi and Di Lisa, 2015; Halestrap and Richardson, 2015). The detailed molecular structure of the mPTP remains unknown. The adenine nucleotide translocase (ANT) and the voltage-dependent anion channel (VDAC) have been proposed as the core mPTP components, however, genetic studies revealed that these proteins play a regulatory rather than a structural role in mPTP induction. Several studies suggested that dimerization of the F_1_F_O_-ATPase (ETC complex V) and the c-subunit ring in the F_O_ domain of the ATP synthase are involved in the mPTP (Giorgio et al., 2013; Alavian et al., 2014). Most recent findings challenged the pore-forming role of the peripheral stalk subunits and the subunit c (He et al., 2017a,b) thus, further studies are required to establish the potential role of the F_1_F_O_-ATPase in mPTP formation. Cyclophilin-D (CyP-D), a matrix located peptidyl-prolyl *cis*–*trans* isomerase, is considered a key mPTP regulator. In addition to mPTP regulation, CyP-D plays an important role in regulating mitochondrial metabolism and bioenergetics under physiological conditions, thereby raising a question whether it is a viable target for cardioprotection (Javadov et al., 2017).

The discovery of the acetylation capacity of CyP-D hinted toward a possible involvement of SIRT3 in modulating mPTP formation. Cardiac-specific deletion of the NDUFS4 subunit of complex I decreased the NAD^+^/NADH ratio, and this was associated with increased mitochondrial protein acetylation and high mPTP sensitivity, thus providing evidence that acetylation could be a mediator of mPTP formation (Karamanlidis et al., 2013). Also, an increase in mitochondrial protein acetylation and reduced SIRT3 expression correlated with increased mPTP opening in a rodent model of post-MI HF (Parodi-Rullan et al., 2012). SIRT3 was shown to interact and deacetylate CyP-D (Hafner et al., 2010) and absence of SIRT3 increased CyP-D acetylation and induced mPTP opening (Hafner et al., 2010; Parodi-Rullan et al., 2012). However, acute oxidative stress (10–30 min) in H9c2 cells did not affect CyP-D acetylation but, instead, further reduced acetyl-CyP-D levels after 60 min of incubation with hydrogen peroxide (Barreto-Torres et al., 2015). Interestingly, mitochondria isolated from SIRT3 deficient hearts demonstrated high basal swelling in the absence of mPTP inducers (IR and Ca^2+^) (Parodi-Rullán et al., 2017). However, another group reported that absence of SIRT3 did not affect the sensitivity of cardiac mitochondria to mPTP (Koentges et al., 2016). The difference observed in the sensitivity of mitochondria to Ca^2+^ in the absence of SIRT3 can be explained by a possible cardioprotective role of hypothermia that was applied to the heart before IR (Koentges et al., 2016). Interestingly, acetylation of CyP-D, and the oligomycin sensitivity conferring protein (OSCP) subunit of the F_1_F_O_-ATP synthase in animals subjected to transverse aortic constriction promoted their interaction inducing mPTP formation, and the interaction was reverted by NAD^+^ (Lee et al., 2016), thus proposing that SIRT3 deacetylation can modulate mPTP induction.

Overexpression of SIRT3 prevented mitochondrial membrane depolarization in triptolide-treated cardiomyocytes, a consequence of mPTP induction (Yang et al., 2016b). Thus, some studies propose that CyP-D acetylation can sensitize mitochondria to mPTP induction and conversely, CyP-D deacetylation by SIRT3 desensitizes mitochondria to mPTP induction, therefore protecting cardiac mitochondria.

### Protein Acetylation in Heart Failure

Heart failure is the final stage after a progressive adaptation of the heart to increased workload induced by MI, hypertension, cardiomyopathy, etc. The failing heart is unable to pump sufficient amount of oxygen and substrates to the organs and tissues, which results in maladaptive secondary alterations. Mice with SIRT3 deficiency display signs of cardiac hypertrophy and interstitial fibrosis by 8 weeks of age. In response to hypertrophic stimuli, SIRT3 deficient mice developed severe cardiac hypertrophy, in contrast to SIRT3-overexpressing mice that did not respond to these stimuli (Sundaresan et al., 2009). In animal models of HF, SIRT3 expression was reduced and acetylation of mitochondrial proteins was increased (Grillon et al., 2012; Parodi-Rullan et al., 2012; Horton et al., 2016; Lee et al., 2016). In addition, the failing hearts contain decreased levels of NAD^+^ (Horton et al., 2016; Lee et al., 2016).

Indeed, several proteins have been found to be acetylated in animal models of HF. Protein acetylation was associated with downregulation of their enzymatic activity, and metabolic alterations in the heart. During normal cardiac physiology, the mitochondria primarily utilizes FAO (up to 90%) as the source of ATP. However, during HF, FAO is reduced, and glucose oxidation is increased, but as HF progresses, the body becomes insulin resistant further compromising cardiac energy metabolism. Impaired fuel utilization is accompanied by energy depletion, with a drop in ATP levels leading to contractile dysfunction (reviewed in Neubauer, 2007). In HF, LCAD was found to be more acetylated which has been associated with decreased activity, and that correlates with the reduction in FAO observed in HF (Grillon et al., 2012). Other groups have reported that SDH activity, respiration rates, and ATP levels were significantly reduced in failing hearts (Horton et al., 2016). Indeed, various enzymes involved in ATP production have been found acetylated in HF (Grillon et al., 2012). Also, in cardiomyocytes from guinea-pigs that developed HF, ROS levels were elevated (Goh et al., 2016) which could be due to a reduction in the activity of the primary antioxidant in the mitochondria, MnSOD, which has been extensively studied as a SIRT3 target.

Likewise, patients with end-stage HF exhibited decreased expression of SIRT3 and increased differentiation of fibroblasts to myofibroblasts, a critical step in developing tissue fibrosis (Sundaresan et al., 2016). Indeed, SIRT3 KO mice developed tissue fibrosis in the heart, liver, kidney, and lungs in an age-dependent manner and this was associated with increased TGF-β1 expression and hyperacetylation of glycogen synthase kinase 3β (GSK-3β). Conversely, SIRT3 overexpression blocked aging-associated tissue fibrosis in mouse cardiac fibroblasts through SIRT3-induced deacetylation and activation of GSK3β which, in turn, inhibited TGF-β1 signaling and prevented tissue fibrosis (Sundaresan et al., 2016). Additionally, in accord to animal models, failing human hearts also displayed increased acetylation of mitochondrial proteins, particularly the enzymes involved in energy metabolism such as IDH and MDH, associated with reduced NAD^+^ levels (Horton et al., 2016).

Thus, SIRT3 acts as a negative regulator of cardiac hypertrophy and aging-associated tissue fibrosis. It is tempting to speculate that the mitochondrial metabolic alterations observed in severe hypertrophy and HF are, at least in part, the result of hyperacetylation of mitochondrial proteins due to reduced activity of SIRT3.

## SIRT3 as a Therapeutic Target for Cardiovascular Diseases

**FIGURE 2:**
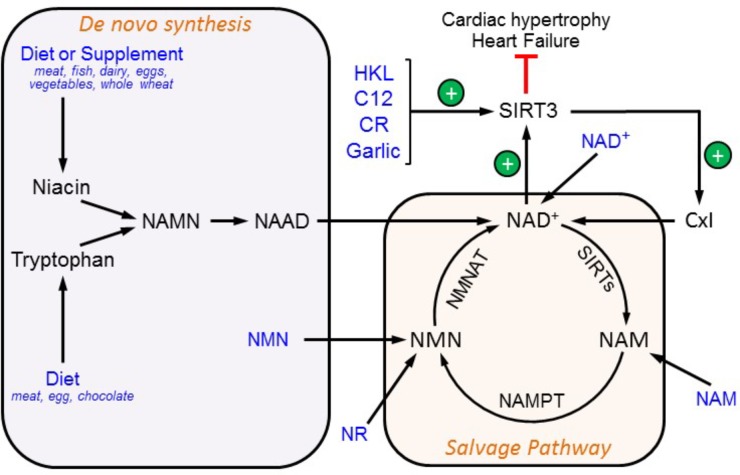
Therapeutic approaches to target SIRT3. SIRT3 activity can be modulated by an increase in NAD^+^ availability and through direct SIRT3 activation. SIRT3 activity prevents cardiac hypertrophy thereby inhibiting the development of HF. Possible nutritional supplements and compounds that increase SIRT3 activation are shown in blue. C12, 7-hydroxyl-3-(4′-metoxyphenyl) coumarin; CR, caloric restriction; HKL, honokiol; NAAD, nicotinic acid adenine dinucleotide; NAMN, nicotinic acid mononucleotide; NAM, nicotinamide; NAMPT, nicotinamide phosphoribosyltransferase; NMN, nicotinamide mononucleotide; NMNAT, nicotinamide mononucleotide adenylyltransferase; NR, nicotinamide ribose.

A growing number of studies portraying SIRT3 as a key regulator of mitochondrial metabolism and energetics highlight the necessity to develop new pharmacological compounds and conditional approaches targeting SIRT3 for treatment of cardiovascular diseases. Therapeutically, the main goal would be to enhance SIRT3 activity and expression. One of the most important therapeutic approaches is aimed at increasing the mitochondrial NAD^+^ levels (Yoshino et al., 2011; Wang et al., 2014) or other SIRT3 activators (**Figure 2**).

Consumption of tryptophan or niacin in the diet stimulates *de novo* NAD^+^ biosynthesis. A diet rich in meat products, diary, and eggs can increase the NAD^+^ availability, thereby activating SIRT3 (Bogan and Brenner, 2008). Another approach to enhance NAD^+^ synthesis is through the salvage pathway. Since NAMPT converts NAM to nicotinamide mononucleotide (NMN) for NAD^+^ biosynthesis in mammalian cells, supplementation with NMN or overexpressing NAMPT would increase NAD^+^ levels. Indeed, cardiac-specific NAMPT overexpression improved cardiac function and prevented hypertrophy and the progression of HF induced by transverse aortic constriction or isoproterenol treatment in animals (Lee et al., 2016). Treatment with NMN recovered the NAD^+^/NADH ratio and decreased the sensitivity of cardiac mitochondria to Ca^2+^-induced stress in mice with complex I deficiency (Karamanlidis et al., 2013). In mice with a high-fat diet (HFD)-induced type 2 diabetes, NMN improved glucose tolerance and enhanced hepatic insulin sensitivity by restoring NAD^+^ levels. Also, the expression of genes involved in oxidative stress, inflammation, and circadian rhythm was restored by NMN treatment (Yoshino et al., 2011). The beneficial effects of NMN were abrogated by EX527, a SIRT1-specific inhibitor, thereby confirming that SIRT1 is involved in mediating the effects of NMN. A 1-week treatment with NMN reversed the metabolic changes observed during age-related muscle pathology such as increased lactate, reduced ATP levels, and decreased expression of mtDNA-encoded OXPHOS genes. However, the effects of NMN were not observed in SIRT1 or NMN adenylyl transferase-1 KO mice (Gomes et al., 2013). According to the mechanism proposed by the authors, NMN increases SIRT1 activity which then stimulated mitochondrial biogenesis through the deacetylation of PGC-1α. The activity or expression of mitochondrial sirtuins was not analyzed in these studies (Yoshino et al., 2011; Gomes et al., 2013).

Also, NAM supplementation for treatment of pellagra (niacin deficiency) could serve as a therapeutic target (Houtkooper and Auwerx, 2012). Although NAM is mostly used as a SIRT inhibitor, its inhibitory effects are short-lasting, most of the NAM is rapidly converted to NAD^+^ (Hwang and Song, 2017). Also, the NAD^+^ precursor, NR, was able to increase the mitochondrial NAD^+^ levels (Cantó et al., 2012; Khan et al., 2014). Currently, NR has been recommended as a nutritional supplement to maintain the high NAD^+^/NADH ratio. Intriguingly, exogenous NAD^+^ seems to be of equal significance; daily NAD^+^ treatment maintained intracellular levels of NAD^+^ and prevented phenylephrine-induced *in vitro* hypertrophy in cultured cardiomyocytes and angiotensin II-induced cardiac hypertrophy *in vivo* in mice (Pillai et al., 2010). Similarly, intravenous administration of NAD^+^ before ischemia protected the rat heart from IR injury (Zhang et al., 2016).

Additionally, several chemical compounds demonstrated the capability to activate SIRT3. SIRT3 activity has been shown to be markedly increased by HKL, a compound isolated from trees in the Magnolia genus (Pillai et al., 2015, 2016, 2017). Also, a recent screening of different compounds led to the discovery of 7-hydroxy-3-(4′-metoxyphenyl) coumarin (C12) which activated SIRT3 in primary cortical astrocytes (Lu et al., 2017). Lastly, oral garlic administration could increase SIRT3 expression and activity in the diabetic heart (Sultana et al., 2016). In addition to chemical compounds and nutrients, dietary interventions such as CR have been proposed to exert its beneficial effects through SIRT3 by increasing its activity or expression (Yang et al., 2007; Hirschey et al., 2010; Shinmura et al., 2011). The precise mechanisms of CR have not been fully understood but can be explained, in part, by increases in the NAD^+/^NADH ratio. Increased NADH levels as a result of overnutrition associated with obesity and diabetes reduces the NAD^+/^NADH ratio. The latter, in turn, increases protein acetylation in mitochondria due to SIRT3 inactivation (Cantó et al., 2012; Alrob et al., 2014). Apparently, similar mechanisms mediate the alterations in the ETC and OXPHOS complexes associated with the decreased NAD^+/^NADH ratio which then stimulate mitochondrial protein acetylation (Wagner et al., 2012; Karamanlidis et al., 2013). Conversely, CR was able to reduce oxidative stress (Qiu et al., 2010) and delay disease onset and mortality (Colman et al., 2009) in animal models. Although the mechanisms underlying SIRT3 activation remain unclear, several lines of therapeutic strategies aiming at increasing SIRT3 activity are available for clinical consideration.

## Conclusion

Numerous studies have provided strong evidence of the involvement of mitochondrial protein acetylation in cardiac pathology therefore portraying SIRT3 as the main regulator of mitochondrial metabolism and function. The main role of SIRT3 is to maintain mitochondrial bioenergetics by regulating the activity of enzymes involved in FAO, TCA cycle, ETC, and OXPHOS in the heart. The heart is the most energy consuming organ and the mitochondria, the main powerhouse of the cell, occupy 30–35% of total cardiomyocyte volume and supply ∼90% of ATP needed for normal cardiac function. Mitochondria also play a central role in cell death and are involved in the pathogenesis of cardiac diseases, particularly, MI, IR, and HF. Therefore, a careful balance between energetic expenditure and generation is important for maintenance of optimal cardiac function. SIRT3 appears to maintain the balance between cardiac function and energy expenditure by regulating the activity of enzymes involved in ATP production in the mitochondria. Downregulation of SIRT3 due to its inhibition or low expression may lead to the imbalance in mitochondrial bioenergetics associated with mitochondrial dysfunction. Therefore, the maintenance of SIRT3 activity is important for normal mitochondrial metabolism and function and this strategy can be useful for the prevention of pathological consequences of cardiac diseases.

## Author Contributions

RP-R reviewed and analyzed the literature, prepared a draft of the manuscript and proofed it. XC-D reviewed and analyzed the literature and participated in writing the manuscript. SJ supervised the project, edited and compiled final version of the manuscript.

## Conflict of Interest Statement

The authors declare that the research was conducted in the absence of any commercial or financial relationships that could be construed as a potential conflict of interest. The reviewer MB and handling Editor declared their shared affiliation.

## Abbreviations

BER: base excision repair
CR: caloric restriction
CyP-D: cyclophilin D
Drp1: dynamin related protein 1
ETC: electron transport chain
FAO: fatty acid oxidation
Fis1: mitochondrial fission 1
FOXO3a: Forkhead box protein O3
HF: heart failure
HKL: honokiol
IDH: isocitrate dehydrogenase
IMM: inner mitochondrial membrane
IR: ischemia-reperfusion
KO: knockout
LCAD: long-chain acyl-CoA dehydrogenase
MDH: malate dehydrogenase
MEFs: mouse embryonic fibroblasts
Mfn1/2: mitofusin 1/2
MI: myocardial infarction
MnSOD: Mg^2+^-dependent mitochondrial superoxide dismutase
mPTP: mitochondrial permeability transition pore
mtDNA: mitochondrial DNA
NAM: nicotinamide
NR: nicotinamide ribose
OMM: outer mitochondrial membrane
OPA1: optic atrophy 1
OXPHOS: oxidative phosphorylation
PDH: pyruvate dehydrogenase
PGC-1α: proliferator-activated receptor gamma coactivator-1 alpha
PINK1: serine/threonine-protein kinase
PTMs: post-translational modifications
ROS: reactive oxygen species
SDH: succinate dehydrogenase
TCA: tricarboxylic acid
WT: wild-type

## References

[B1] Abdel Khalek W. Cortade F. Ollendorff V. Lapasset L. Tintignac L. Chabi B. (2014). SIRT3, a mitochondrial NAD?-dependent deacetylase, is involved in the regulation of myoblast differentiation. *PLoS One* 9:e114388. 10.1371/journal.pone.0114388 25489948 PMC4260865

[B2] Abo Alrob O. Lopaschuk G. D. (2014). Role of CoA and acetyl-CoA in regulating cardiac fatty acid and glucose oxidation. *Biochem. Soc. Trans.* 42 1043–1051. 10.1042/BST20140094 25110000

[B3] Ahn B.-H. Kim H. S. Song S. Lee I. H. Liu J. Vassilopoulos A. (2008). A role for the mitochondrial deacetylase Sirt3 in regulating energy homeostasis. *Proc. Natl. Acad. Sci. U.S.A.* 105 14447–14452. 10.1073/pnas.0803790105 18794531 PMC2567183

[B4] Ahuja N. Schwer B. Carobbio S. Waltregny D. North B. J. Castronovo V. (2007). Regulation of insulin secretion by SIRT4, a mitochondrial ADP-ribosyltransferase. *J. Biol. Chem.* 282 33583–33592. 10.1074/jbc.M705488200 17715127

[B5] Alavian K. N. Beutner G. Lazrove E. Sacchetti S. Park H.-A. Licznerski P. (2014). An uncoupling channel within the c-subunit ring of the F1FO ATP synthase is the mitochondrial permeability transition pore. *Proc. Natl. Acad. Sci. U.S.A.* 111 10580–10585. 10.1073/pnas.1401591111 24979777 PMC4115574

[B6] Alcendor R. R. Gao S. Zhai P. Zablocki D. Holle E. Yu X. (2007). Sirt1 regulates aging and resistance to oxidative stress in the heart. *Circ. Res.* 100 1512–1521. 10.1161/01.RES.0000267723.65696.4a 17446436

[B7] Alexeyev M. Shokolenko I. Wilson G. LeDoux S. (2013). The maintenance of mitochondrial DNA integrity–critical analysis and update. *Cold Spring Harb. Perspect. Biol.* 5:a012641. 10.1101/cshperspect.a012641 23637283 PMC3632056

[B8] Alrob O. A. Sankaralingam S. Ma C. Wagg C. S. Fillmore N. Jaswal J. S. (2014). Obesity-induced lysine acetylation increases cardiac fatty acid oxidation and impairs insulin signalling. *Cardiovasc. Res.* 103 485–497. 10.1093/cvr/cvu156 24966184 PMC4155471

[B9] Araki S. Izumiya Y. Rokutanda T. Ianni A. Hanatani S. Kimura Y. (2015). Sirt7 contributes to myocardial tissue repair by maintaining transforming growth factor-β signaling pathway. *Circulation* 132 1081–1093. 10.1161/CIRCULATIONAHA.114.014821 26202810

[B10] Arora A. Dey C. S. (2014). SIRT2 negatively regulates insulin resistance in C2C12 skeletal muscle cells. *Biochim. Biophys. Acta* 1842 1372–1378. 10.1016/j.bbadis.2014.04.027 24793418

[B11] Ashrafi G. Schwarz T. L. (2013). The pathways of mitophagy for quality control and clearance of mitochondria. *Cell Death Differ.* 20 31–42. 10.1038/cdd.2012.81 22743996 PMC3524633

[B12] Bae N. S. Swanson M. J. Vassilev A. Howard B. H. (2004). Human histone deacetylase SIRT2 interacts with the homeobox transcription factor HOXA10. *J. Biochem.* 135 695–700. 10.1093/jb/mvh084 15213244

[B13] Baeza J. Smallegan M. J. Denu J. M. (2015). Site-specific reactivity of nonenzymatic lysine acetylation. *ACS Chem. Biol.* 10 122–128. 10.1021/cb500848p 25555129 PMC4301072

[B14] Baeza J. Smallegan M. J. Denu J. M. (2016). Mechanisms and dynamics of protein acetylation in mitochondria. *Trends Biochem. Sci.* 41 231–244. 10.1016/j.tibs.2015.12.006 26822488 PMC4783225

[B15] Barreto-Torres G. Hernandez J. S. Jang S. Rodríguez-Muñoz A. R. Torres-Ramos C. A. Basnakian A. G. (2015). The beneficial effects of AMP kinase activation against oxidative stress are associated with prevention of PPARα-cyclophilin D interaction in cardiomyocytes. *Am. J. Physiol. Heart Circ. Physiol.* 308 H749–H758. 10.1152/ajpheart.00414.2014 25617357 PMC4385997

[B16] Bellizzi D. Rose G. Cavalcante P. Covello G. Dato S. De Rango F. (2005). A novel VNTR enhancer within the SIRT3 gene, a human homologue of SIR2, is associated with survival at oldest ages. *Genomics* 85 258–263. 10.1016/j.ygeno.2004.11.003 15676284

[B17] Bernardi P. Di Lisa F. (2015). The mitochondrial permeability transition pore: molecular nature and role as a target in cardioprotection. *J. Mol. Cell. Cardiol.* 78 100–106. 10.1016/j.yjmcc.2014.09.023 25268651 PMC4294587

[B18] Bharathi S. S. Zhang Y. Mohsen A. W. Uppala R. Balasubramani M. Schreiber E. (2013). Sirtuin 3 (SIRT3) protein regulates long-chain acyl-CoA dehydrogenase by deacetylating conserved lysines near the active site. *J. Biol. Chem.* 288 33837–33847. 10.1074/jbc.M113.510354 24121500 PMC3837126

[B19] Bindu S. Pillai V. B. Gupta M. P. (2016). Role of sirtuins in regulating pathophysiology of the heart. *Trends Endocrinol. Metab.* 27 563–573. 10.1016/j.tem.2016.04.015 27210897

[B20] Bogan K. L. Brenner C. (2008). Nicotinic acid, nicotinamide, and nicotinamide riboside: a molecular evaluation of NAD+ precursor vitamins in human nutrition. *Annu. Rev. Nutr.* 28 115–130. 10.1146/annurev.nutr.28.061807.155443 18429699

[B21] Borniquel S. García-Quintáns N. Valle I. Olmos Y. Wild B. Martínez-Granero F. (2010). Inactivation of Foxo3a and subsequent downregulation of PGC-1 alpha mediate nitric oxide-induced endothelial cell migration. *Mol. Cell. Biol.* 30 4035–4044. 10.1128/MCB.00175-10 20547753 PMC2916439

[B22] Boylston J. A. Sun J. Chen Y. Gucek M. Sack M. N. Murphy E. (2015). Characterization of the cardiac succinylome and its role in ischemia–reperfusion injury. *J. Mol. Cell. Cardiol.* 88 73–81. 10.1016/j.yjmcc.2015.09.005 26388266 PMC4780049

[B23] Brand M. D. (2010). The sites and topology of mitochondrial superoxide production. *Exp. Gerontol.* 45 466–472. 10.1016/j.exger.2010.01.003 20064600 PMC2879443

[B24] Bugger H. Witt C. N. Bode C. (2016). Mitochondrial sirtuins in the heart. *Heart Fail. Rev.* 21 519–528. 10.1007/s10741-016-9570-7 27295248

[B25] Cantó C. Houtkooper R. H. Pirinen E. Youn D. Y. Oosterveer M. H. Cen Y. (2012). The NAD+ precursor nicotinamide riboside enhances oxidative metabolism and protects against high-fat diet-induced obesity. *Cell Metab.* 15 838–847. 10.1016/j.cmet.2012.04.022 22682224 PMC3616313

[B26] Chen C. J. Fu Y. C. Yu W. Wang W. (2013). SIRT3 protects cardiomyocytes from oxidative stress-mediated cell death by activating NF-κB. *Biochem. Biophys. Res. Commun.* 430 798–803. 10.1016/j.bbrc.2012.11.066 23201401

[B27] Chen L. Gong Q. Stice J. P. Knowlton A. A. (2009). Mitochondrial OPA1, apoptosis, and heart failure. *Cardiovasc. Res.* 84 91–99. 10.1093/cvr/cvp181 19493956 PMC2741347

[B28] Chen L. Liu T. Tran A. Lu X. Tomilov A. A. Davies V. (2012). OPA1 mutation and late-onset cardiomyopathy: mitochondrial dysfunction and mtDNA instability. *J. Am. Heart Assoc.* 1:e003012. 10.1161/JAHA.112.003012 23316298 PMC3541627

[B29] Chen T. Liu J. Li N. Wang S. Liu H. Li J. (2015). Mouse SIRT3 attenuates hypertrophy-related lipid accumulation in the heart through the deacetylation of LCAD. *PLoS One* 10:e0118909. 10.1371/journal.pone.0118909 25748450 PMC4351969

[B30] Chen Y. Liu Y. Dorn G. W. (2011). Mitochondrial fusion is essential for organelle function and cardiac homeostasis. *Circ. Res.* 109 1327–1331. 10.1161/CIRCRESAHA.111.258723 22052916 PMC3237902

[B31] Cimen H. Han M. J. Yang Y. Tong Q. Koc H. Koc E. C. (2010). Regulation of succinate dehydrogenase activity by SIRT3 in mammalian mitochondria. *Biochemistry* 49 304–311. 10.1021/bi901627u 20000467 PMC2826167

[B32] Colman R. J. Anderson R. M. Johnson S. C. Kastman E. K. Kosmatka K. J. Beasley T. M. (2009). Caloric restriction delays disease onset and mortality in rhesus monkeys. *Science* 325 201–204. 10.1126/science.1173635 19590001 PMC2812811

[B33] Dittenhafer-Reed K. E. Richards A. L. Fan J. Smallegan M. J. Fotuhi Siahpirani A. Kemmerer Z. A. (2015). SIRT3 mediates multi-tissue coupling for metabolic fuel switching. *Cell Metab.* 21 637–646. 10.1016/j.cmet.2015.03.007 25863253 PMC4393847

[B34] Dryden S. C. Nahhas F. A. Nowak J. E. Goustin A.-S. Tainsky M. A. (2003). Role for human SIRT2 NAD-dependent deacetylase activity in control of mitotic exit in the cell cycle. *Mol. Cell. Biol.* 23 3173–3185. 10.1128/MCB.23.9.3173-3185.2003 12697818 PMC153197

[B35] Du J. Zhou Y. Su X. Yu J. J. Khan S. Jiang H. (2011). Sirt5 is a NAD-dependent protein lysine demalonylase and desuccinylase. *Science* 334 806–809. 10.1126/science.1207861 22076378 PMC3217313

[B36] Fan J. Shan C. Kang H.-B. Elf S. Xie J. Tucker M. (2014). Tyr phosphorylation of PDP1 toggles recruitment between ACAT1 and SIRT3 to regulate the pyruvate dehydrogenase complex. *Mol. Cell* 53 534–548. 10.1016/j.molcel.2013.12.026 24486017 PMC3943932

[B37] Fan W. Evans R. (2015). PPARs and ERRs: molecular mediators of mitochondrial metabolism. *Curr. Opin. Cell Biol.* 33 49–54. 10.1016/j.ceb.2014.11.002 25486445 PMC4380823

[B38] Fang L. Moore X.-L. Gao X.-M. Dart A. M. Lim Y. L. Du X.-J. (2007). Down-regulation of mitofusin-2 expression in cardiac hypertrophy in vitro and in vivo. *Life Sci.* 80 2154–2160. 10.1016/j.lfs.2007.04.003 17499311

[B39] Fernandes J. Weddle A. Kinter C. S. Humphries K. M. Mather T. Szweda L. I. (2015). Lysine acetylation activates mitochondrial aconitase in the heart. *Biochemistry* 54 4008–4018. 10.1021/acs.biochem.5b00375 26061789 PMC4513942

[B40] Fernandez-Marcos P. J. Jeninga E. H. Cantó C. Harach T. de Boer V. C. J. Andreux P. (2012). Muscle or liver-specific Sirt3 deficiency induces hyperacetylation of mitochondrial proteins without affecting global metabolic homeostasis. *Sci. Rep.* 2:425. 10.1038/srep00425 22645641 PMC3361023

[B41] Finck B. N. Kelly D. P. (2007). Peroxisome proliferator-activated receptor gamma coactivator-1 (PGC-1) regulatory cascade in cardiac physiology and disease. *Circulation* 115 2540–2548. 10.1161/CIRCULATIONAHA.107.670588 17502589

[B42] Finley L. W. S. Haas W. Desquiret-Dumas V. Wallace D. C. Procaccio V. Gygi S. P. (2011). Succinate dehydrogenase is a direct target of sirtuin 3 deacetylase activity. *PLoS One* 6:e23295. 10.1371/journal.pone.0023295 21858060 PMC3157345

[B43] Foster D. B. Liu T. Rucker J. O’Meally R. N. Devine L. R. Cole R. N. (2013). The cardiac acetyl-lysine proteome. *PLoS One* 8:e67513. 10.1371/journal.pone.0067513 23844019 PMC3699649

[B44] Frey N. Olson E. N. (2003). Cardiac hypertrophy: the good, the bad, and the ugly. *Annu. Rev. Physiol.* 65 45–79. 10.1146/annurev.physiol.65.092101.142243 12524460

[B45] Frye R. A. (1999). Characterization of five human cDNAs with homology to the yeast SIR2 gene: Sir2-like proteins (sirtuins) metabolize NAD and may have protein ADP-ribosyltransferase activity. *Biochem. Biophys. Res. Commun.* 260 273–279. 10.1006/bbrc.1999.0897 10381378

[B46] Gao J. Zheng Z. Gu Q. Chen X. Liu X. Xu X. (2016). Deacetylation of MnSOD by PARP-regulated SIRT3 protects retinal capillary endothelial cells from hyperglycemia-induced damage. *Biochem. Biophys. Res. Commun.* 472 425–431. 10.1016/j.bbrc.2015.12.037 26692487

[B47] Garnier A. Fortin D. Deloménie C. Momken I. Veksler V. Ventura-Clapier R. (2003). Depressed mitochondrial transcription factors and oxidative capacity in rat failing cardiac and skeletal muscles. *J. Physiol.* 551 491–501. 10.1113/jphysiol.2003.045104 12824444 PMC2343221

[B48] Giorgio V. von Stockum S. Antoniel M. Fabbro A. Fogolari F. Forte M. (2013). Dimers of mitochondrial ATP synthase form the permeability transition pore. *Proc. Natl. Acad. Sci. U.S.A.* 110 5887–5892. 10.1073/pnas.1217823110 23530243 PMC3625323

[B49] Glozak M. A. Sengupta N. Zhang X. Seto E. (2005). Acetylation and deacetylation of non-histone proteins. *Gene* 363 15–23. 10.1016/j.gene.2005.09.010 16289629

[B50] Goh K. Y. Qu J. Hong H. Liu T. Dell’Italia L. J. Wu Y. (2016). Impaired mitochondrial network excitability in failing guinea-pig cardiomyocytes. *Cardiovasc. Res.* 109 79–89. 10.1093/cvr/cvv230 26433944 PMC4692289

[B51] Gomes A. P. Price N. L. Ling A. J. Y. Moslehi J. J. Montgomery M. K. Rajman L. (2013). Declining NAD(+) induces a pseudohypoxic state disrupting nuclear-mitochondrial communication during aging. *Cell* 155 1624–1638. 10.1016/j.cell.2013.11.037 24360282 PMC4076149

[B52] Gomes P. Fleming Outeiro T. Cavadas C. (2015). Emerging Role of sirtuin 2 in the regulation of mammalian metabolism. *Trends Pharmacol. Sci.* 36 756–768. 10.1016/j.tips.2015.08.001 26538315

[B53] Gray S. G. Ekström T. J. (2001). The human histone deacetylase family. *Exp. Cell Res.* 262 75–83. 10.1006/excr.2000.5080 11139331

[B54] Grillon J. M. Johnson K. R. Kotlo K. Danziger R. S. (2012). Non-histone lysine acetylated proteins in heart failure. *Biochim. Biophys. Acta* 1822 607–614. 10.1016/j.bbadis.2011.11.016 22155497 PMC3684243

[B55] Gu W. Roeder R. G. (1997). Activation of p53 sequence-specific DNA binding by acetylation of the p53 C-terminal domain. *Cell* 90 595–606. 10.1016/S0092-8674(00)80521-8 9288740

[B56] Guan K. L. Xiong Y. (2011). Regulation of intermediary metabolism by protein acetylation. *Trends Biochem. Sci.* 36 108–116. 10.1016/j.tibs.2010.09.003 20934340 PMC3038179

[B57] Hafner A. V. Dai J. Gomes A. P. Xiao C.-Y. Palmeira C. M. Rosenzweig A. (2010). Regulation of the mPTP by SIRT3-mediated deacetylation of CypD at lysine 166 suppresses age-related cardiac hypertrophy. *Aging* 2 914–923. 10.18632/aging.100252 21212461 PMC3034180

[B58] Hagenbuchner J. Ausserlechner M. J. (2013). Mitochondria and FOXO3: breath or die. *Front. Physiol.* 4:147 10.3389/fphys.2013.00147 PMC3687139 23801966

[B59] Halestrap A. P. Richardson A. P. (2015). The mitochondrial permeability transition: a current perspective on its identity and role in ischaemia/reperfusion injury. *J. Mol. Cell. Cardiol.* 78 129–141. 10.1016/j.yjmcc.2014.08.018 25179911

[B60] Hallows W. C. Lee S. Denu J. M. (2006). Sirtuins deacetylate and activate mammalian acetyl-CoA synthetases. *Proc. Natl. Acad. Sci. U.S.A.* 103 10230–10235. 10.1073/pnas.0604392103 16790548 PMC1480596

[B61] Hallows W. C. Yu W. Smith B. C. Devries M. K. Devires M. K. Ellinger J. J. (2011). Sirt3 promotes the urea cycle and fatty acid oxidation during dietary restriction. *Mol. Cell* 41 139–149. 10.1016/j.molcel.2011.01.002 21255725 PMC3101115

[B62] Han C. Someya S. (2013). Maintaining good hearing: calorie restriction, Sirt3, and glutathione. *Exp. Gerontol.* 48 1091–1095. 10.1016/j.exger.2013.02.014 23454634 PMC3759555

[B63] He J. Carroll J. Ding S. Fearnley I. M. Walker J. E. (2017a). Permeability transition in human mitochondria persists in the absence of peripheral stalk subunits of ATP synthase. *Proc. Natl. Acad. Sci. U.S.A.* 114 9086–9091. 10.1073/pnas.1711201114 28784775 PMC5576841

[B64] He J. Ford H. C. Carroll J. Ding S. Fearnley I. M. Walker J. E. (2017b). Persistence of the mitochondrial permeability transition in the absence of subunit c of human ATP synthase. *Proc. Natl. Acad. Sci. U.S.A.* 114 3409–3414. 10.1073/pnas.1702357114 28289229 PMC5380099

[B65] Hebert A. S. Dittenhafer-Reed K. E. Yu W. Bailey D. J. Selen E. S. Boersma M. D. (2013). Calorie restriction and SIRT3 trigger global reprogramming of the mitochondrial protein acetylome. *Mol. Cell* 49 186–199. 10.1016/j.molcel.2012.10.024 23201123 PMC3704155

[B66] Hernández J. S. Barreto-Torres G. Kuznetsov A. V. Khuchua Z. Javadov S. (2014). Crosstalk between AMPK activation and angiotensin II-induced hypertrophy in cardiomyocytes: the role of mitochondria. *J. Cell. Mol. Med.* 18 709–720. 10.1111/jcmm.12220 24444314 PMC3981893

[B67] Hirschey M. D. Shimazu T. Goetzman E. Jing E. Schwer B. Lombard D. B. (2010). SIRT3 regulates mitochondrial fatty-acid oxidation by reversible enzyme deacetylation. *Nature* 464 121–125. 10.1038/nature08778 20203611 PMC2841477

[B68] Hirschey M. D. Shimazu T. Jing E. Grueter C. A. Collins A. M. Aouizerat B. (2011). SIRT3 deficiency and mitochondrial protein hyperacetylation accelerate the development of the metabolic syndrome. *Mol. Cell* 44 177–190. 10.1016/j.molcel.2011.07.019 21856199 PMC3563434

[B69] Horton J. L. Martin O. J. Lai L. Riley N. M. Richards A. L. Vega R. B. (2016). Mitochondrial protein hyperacetylation in the failing heart. *JCI Insight* 1 1–14. 10.1172/jci.insight.84897 26998524 PMC4795836

[B70] Houtkooper R. H. Auwerx J. (2012). Exploring the therapeutic space around NAD+. *J. Cell Biol.* 199 205–209. 10.1083/jcb.201207019 23071150 PMC3471225

[B71] Hwang E. S. Song S. B. (2017). Nicotinamide is an inhibitor of SIRT1 in vitro, but can be a stimulator in cells. *Cell. Mol. Life Sci.* 74 3347–3362. 10.1007/s00018-017-2527-8 28417163 PMC11107671

[B72] Jacobs K. M. Pennington J. D. Bisht K. S. Aykin-Burns N. Kim H. S. Mishra M. (2008). SIRT3 interacts with the daf-16 homolog FOXO3a in the mitochondria, as well as increases FOXO3a dependent gene expression. *Int. J. Biol. Sci.* 4 291–299. 10.7150/ijbs.4.291 18781224 PMC2532794

[B73] Jang S.-Y. Kang H. T. Hwang E. S. (2012). Nicotinamide-induced mitophagy: event mediated by high NAD+/NADH ratio and SIRT1 protein activation. *J. Biol. Chem.* 287 19304–19314. 10.1074/jbc.M112.363747 22493485 PMC3365962

[B74] Javadov S. Jang S. Parodi-Rullan R. Khuchua Z. Kuznetsov A. V. (2017). Mitochondrial permeability transition in cardiac ischemia–reperfusion: Whether cyclophilin D is a viable target for cardioprotection? *Cell. Mol. Life Sci.* 74 2795–2813. 10.1007/s00018-017-2502-4 28378042 PMC5977999

[B75] Javadov S. Karmazyn M. Escobales N. (2009). Mitochondrial permeability transition pore opening as a promising therapeutic target in cardiac diseases. *J. Pharmacol. Exp. Ther.* 330 670–678. 10.1124/jpet.109.153213 19509316

[B76] Javadov S. Purdham D. M. Zeidan A. Karmazyn M. (2006). NHE-1 inhibition improves cardiac mitochondrial function through regulation of mitochondrial biogenesis during postinfarction remodeling. *Am. J. Physiol. Heart Circ. Physiol.* 291 H1722–H1730. 10.1152/ajpheart.00159.2006 16679399

[B77] Javadov S. Rajapurohitam V. Kiliæ A. Hunter J. C. Zeidan A. Said Faruq N. (2011). Expression of mitochondrial fusion-fission proteins during post-infarction remodeling: the effect of NHE-1 inhibition. *Basic Res. Cardiol.* 106 99–109. 10.1007/s00395-010-0122-3 20886221

[B78] Jin S. M. Youle R. J. (2012). PINK1- and Parkin-mediated mitophagy at a glance. *J. Cell Sci.* 125 795–799. 10.1242/jcs.093849 22448035 PMC3656616

[B79] Jing E. O’Neill B. T. Rardin M. J. Kleinridders A. Ilkeyeva O. R. Ussar S. (2013). Sirt3 regulates metabolic flexibility of skeletal muscle through reversible enzymatic deacetylation. *Diabetes Metab. Res. Rev.* 62 3404–3417. 10.2337/db12-1650 23835326 PMC3781465

[B80] Kang H. T. Hwang E. S. (2009). Nicotinamide enhances mitochondria quality through autophagy activation in human cells. *Aging Cell* 8 426–438. 10.1111/j.1474-9726.2009.00487.x 19473119

[B81] Karamanlidis G. Lee C. F. Garcia-Menendez L. Kolwicz S. C. Suthammarak W. Gong G. (2013). Mitochondrial complex I deficiency increases protein acetylation and accelerates heart failure. *Cell Metab.* 18 239–250. 10.1016/j.cmet.2013.07.002 23931755 PMC3779647

[B82] Khan N. A. Auranen M. Paetau I. Pirinen E. Euro L. Forsström S. (2014). Effective treatment of mitochondrial myopathy by nicotinamide riboside, a vitamin B3. *EMBO Mol. Med.* 6 721–731. 10.1002/emmm.201403943 24711540 PMC4203351

[B83] Kim S. C. Sprung R. Chen Y. Xu Y. Ball H. Pei J. (2006). Substrate and functional diversity of lysine acetylation revealed by a proteomics survey. *Mol. Cell* 23 607–618. 10.1016/j.molcel.2006.06.026 16916647

[B84] Kiran S. Chatterjee N. Singh S. Kaul S. C. Wadhwa R. Ramakrishna G. (2013). Intracellular distribution of human SIRT7 and mapping of the nuclear/nucleolar localization signal. *FEBS J.* 280 3451–3466. 10.1111/febs.12346 23680022

[B85] Koentges C. Pfeil K. Meyer-Steenbuck M. Lother A. Hoffmann M. M. Odening K. E. (2016). Preserved recovery of cardiac function following ischemia–reperfusion in mice lacking SIRT3. *Can. J. Physiol. Pharmacol.* 94 72–80. 10.1139/cjpp-2015-0152 26524632

[B86] Koentges C. Pfeil K. Schnick T. Wiese S. Dahlbock R. Cimolai M. C. (2015). SIRT3 deficiency impairs mitochondrial and contractile function in the heart. *Basic Res. Cardiol.* 110:36. 10.1007/s00395-015-0493-6 25962702

[B87] Kong X. Wang R. Xue Y. Liu X. Zhang H. Chen Y. (2010). Sirtuin 3, a new target of PGC-1alpha, plays an important role in the suppression of ROS and mitochondrial biogenesis. *PLoS One* 5:e11707. 10.1371/journal.pone.0011707 20661474 PMC2908542

[B88] Laurent G. German N. J. Saha A. K. de Boer V. C. J. Davies M. Koves T. R. (2013). SIRT4 coordinates the balance between lipid synthesis and catabolism by repressing malonyl CoA decarboxylase. *Mol. Cell* 50 686–698. 10.1016/j.molcel.2013.05.012 23746352 PMC3721068

[B89] Lee C. F. Chavez J. D. Garcia-Menendez L. Choi Y. Roe N. D. Chiao Y. A. (2016). Normalization of NAD+ redox balance as a therapy for heart failure. *Circulation* 134 883–894. 10.1161/CIRCULATIONAHA.116.022495 27489254 PMC5193133

[B90] Liszt G. Ford E. Kurtev M. Guarente L. (2005). Mouse Sir2 homolog SIRT6 is a nuclear ADP-ribosyltransferase. *J. Biol. Chem.* 280 21313–21320. 10.1074/jbc.M413296200 15795229

[B91] Liu B. Che W. Xue J. Zheng C. Tang K. Zhang J. (2013a). SIRT4 prevents hypoxia-induced apoptosis in H9c2 cardiomyoblast cells. *Cell. Physiol. Biochem.* 32 655–662. 10.1159/000354469 24029877

[B92] Liu B. Che W. Zheng C. Liu W. Wen J. Fu H. (2013b). SIRT5: a safeguard against oxidative stress-induced apoptosis in cardiomyocytes. *Cell. Physiol. Biochem.* 32 1050–1059. 10.1159/000354505 24192575

[B93] Liu G. Park S. H. Imbesi M. Nathan W. J. Zou X. Zhu Y. (2017). Loss of NAD-dependent protein deacetylase sirtuin-2 alters mitochondrial protein acetylation and dysregulates mitophagy. *Antioxid. Redox Signal.* 26 846–863. 10.1089/ars.2016.6662 27460777 PMC5444513

[B94] Lombard D. B. Alt F. W. Cheng H. L. Bunkenborg J. Streeper R. S. Mostoslavsky R. (2007). Mammalian Sir2 homolog SIRT3 regulates global mitochondrial lysine acetylation. *Mol. Cell. Biol.* 27 8807–8814. 10.1128/MCB.01636-07 17923681 PMC2169418

[B95] Long Q. Yang K. Yang Q. (2015). Regulation of mitochondrial ATP synthase in cardiac pathophysiology. *Am. J. Cardiovasc. Dis.* 5 19–32. 26064790 PMC4447074

[B96] Lu J. Zhang H. Chen X. Zou Y. Li J. Wang L. (2017). A small molecule activator of SIRT3 promotes deacetylation and activation of manganese superoxide dismutase. *Free Radic. Biol. Med.* 112 287–297. 10.1016/j.freeradbiomed.2017.07.012 28711502

[B97] Lundby A. Lage K. Weinert B. T. Bekker-Jensen D. B. Secher A. Skovgaard T. (2012). Proteomic analysis of lysine acetylation sites in rat tissues reveals organ specificity and subcellular patterns. *Cell Rep.* 2 419–431. 10.1016/j.celrep.2012.07.006 22902405 PMC4103158

[B98] Luo J. Nikolaev A. Y. Imai S. Chen D. Su F. Shiloh A. (2001). Negative control of p53 by Sir2alpha promotes cell survival under stress. *Cell* 107 137–148. 10.1016/S0092-8674(01)00524-4 11672522

[B99] Luo Y.-X. Tang X. An X.-Z. Xie X.-M. Chen X.-F. Zhao X. (2017). SIRT4 accelerates Ang II-induced pathological cardiac hypertrophy by inhibiting manganese superoxide dismutase activity. *Eur. Heart J.* 38 1389–1398. 10.1093/eurheartj/ehw138 27099261

[B100] Lynn E. G. McLeod C. J. Gordon J. P. Bao J. Sack M. N. (2008). SIRT2 is a negative regulator of anoxia-reoxygenation tolerance via regulation of 14-3-3 zeta and BAD in H9c2 cells. *FEBS Lett.* 582 2857–2862. 10.1016/j.febslet.2008.07.016 18640115 PMC2566947

[B101] Maxwell M. M. Tomkinson E. M. Nobles J. Wizeman J. W. Amore A. M. Quinti L. (2011). The Sirtuin 2 microtubule deacetylase is an abundant neuronal protein that accumulates in the aging CNS. *Hum. Mol. Genet.* 20 3986–3996. 10.1093/hmg/ddr326 21791548 PMC3203628

[B102] Michishita E. McCord R. A. Berber E. Kioi M. Padilla-Nash H. Damian M. (2008). SIRT6 is a histone H3 lysine 9 deacetylase that modulates telomeric chromatin. *Nature* 452 492–496. 10.1038/nature06736 18337721 PMC2646112

[B103] Michishita E. McCord R. A. Boxer L. D. Barber M. F. Hong T. Gozani O. (2009). Cell cycle-dependent deacetylation of telomeric histone H3 lysine K56 by human SIRT6. *Cell Cycle* 8 2664–2666. 10.4161/cc.8.16.9367 19625767 PMC4474138

[B104] Michishita E. Park J. Y. Burneskis J. M. Barrett J. C. Horikawa I. (2005). Evolutionarily conserved and nonconserved cellular localizations and functions of human SIRT proteins. *Mol. Biol. Cell* 16 4623–4635. 10.1091/mbc.E05-01-0033 16079181 PMC1237069

[B105] Mistry N. F. Cresci S. (2010). PPAR transcriptional activator complex polymorphisms and the promise of individualized therapy for heart failure. *Heart Fail. Rev.* 15 197–207. 10.1007/s10741-008-9114-x 18998207 PMC3799956

[B106] Mori J. Alrob O. A. Wagg C. S. Harris R. A. Lopaschuk G. D. Oudit G. Y. (2013). ANG II causes insulin resistance and induces cardiac metabolic switch and inefficiency: a critical role of PDK4. *Am. J. Physiol. Heart Circ. Physiol.* 304 H1103–H1113. 10.1152/ajpheart.00636.2012 23396452

[B107] Mostoslavsky R. Chua K. F. Lombard D. B. Pang W. W. Fischer M. R. Gellon L. (2006). Genomic instability and aging-like phenotype in the absence of mammalian SIRT6. *Cell* 124 315–329. 10.1016/j.cell.2005.11.044 16439206

[B108] Nadtochiy S. M. Redman E. Rahman I. Brookes P. S. (2011). Lysine deacetylation in ischaemic preconditioning: the role of SIRT1. *Cardiovasc. Res.* 89 643–649. 10.1093/cvr/cvq287 20823277 PMC3028968

[B109] Nadtochiy S. M. Wang Y. T. Zhang J. Nehrke K. Schafer X. Welle K. (2017). Potential mechanisms linking SIRT activity and hypoxic 2-hydroxyglutarate generation: no role for direct enzyme (de)acetylation. *Biochem. J.* 474 2829–2839. 10.1042/BCJ20170389 28673962 PMC5562404

[B110] Nakagawa T. Lomb D. J. Haigis M. C. Guarente L. (2009). SIRT5 Deacetylates carbamoyl phosphate synthetase 1 and regulates the urea cycle. *Cell* 137 560–570. 10.1016/j.cell.2009.02.026 19410549 PMC2698666

[B111] Nasrin N. Wu X. Fortier E. Feng Y. Bare O. C. Chen S. (2010). SIRT4 regulates fatty acid oxidation and mitochondrial gene expression in liver and muscle cells. *J. Biol. Chem.* 285 31995–32002. 10.1074/jbc.M110.124164 20685656 PMC2952200

[B112] Neubauer S. (2007). The failing heart–an engine out of fuel. *N. Engl. J. Med.* 356 1140–1151. 10.1056/NEJMra063052 17360992

[B113] Nishida Y. Rardin M. J. Carrico C. He W. Sahu A. K. Gut P. (2015). SIRT5 regulates both cytosolic and mitochondrial protein malonylation with glycolysis as a major target. *Mol. Cell* 59 321–332. 10.1016/j.molcel.2015.05.022 26073543 PMC4571487

[B114] North B. J. Marshall B. L. Borra M. T. Denu J. M. Verdin E. (2003). The human Sir2 ortholog, SIRT2, is an NAD+-dependent tubulin deacetylase. *Mol. Cell* 11 437–444. 10.1016/S1097-2765(03)00038-8 12620231

[B115] Okatsu K. Oka T. Iguchi M. Imamura K. Kosako H. Tani N. (2012). PINK1 autophosphorylation upon membrane potential dissipation is essential for Parkin recruitment to damaged mitochondria. *Nat. Commun.* 3:1016. 10.1038/ncomms2016 22910362 PMC3432468

[B116] Olmos Y. Valle I. Borniquel S. Tierrez A. Soria E. Lamas S. (2009). Mutual dependence of Foxo3a and PGC-1alpha in the induction of oxidative stress genes. *J. Biol. Chem.* 284 14476–14484. 10.1074/jbc.M807397200 19324885 PMC2682896

[B117] Ozden O. Park S. H. Wagner B. A. Song H. Y. Zhu Y. Vassilopoulos A. (2014). SIRT3 deacetylates and increases pyruvate dehydrogenase activity in cancer cells. *Free Radic. Biol. Med.* 76 163–172. 10.1016/j.freeradbiomed.2014.08.001 25152236 PMC4364304

[B118] Palacios O. M. Carmona J. J. Michan S. Chen K. Y. Manabe Y. Ward J. L. (2009). Diet and exercise signals regulate SIRT3 and activate AMPK and PGC-1alpha in skeletal muscle. *Aging* 1 771–783. 10.18632/aging.100075 20157566 PMC2815736

[B119] Park J. Chen Y. Tishkoff D. X. Peng C. Tan M. Dai L. (2013). SIRT5-mediated lysine desuccinylation impacts diverse metabolic pathways. *Mol. Cell* 50 919–930. 10.1016/j.molcel.2013.06.001 23806337 PMC3769971

[B120] Parodi-Rullan R. Barreto-Torres G. Ruiz L. Casasnovas J. Javadov S. (2012). Direct renin inhibition exerts an anti-hypertrophic effect associated with improved mitochondrial function in post-infarction heart failure in diabetic rats. *Cell. Physiol. Biochem.* 29 841–850. 10.1159/000178526 22613984 PMC3980674

[B121] Parodi-Rullán R. M. Chapa-Dubocq X. Rullán P. J. Jang S. Javadov S. (2017). High sensitivity of SIRT3 deficient hearts to ischemia-reperfusion is associated with mitochondrial abnormalities. *Front. Pharmacol.* 8:275 10.3389/fphar.2017.00275 PMC5432544 28559847

[B122] Pillai V. B. Bindu S. Sharp W. Fang Y. H. Kim G. Gupta M. (2016). Sirt3 protects mitochondrial DNA damage and blocks the development of doxorubicin-induced cardiomyopathy in mice. *Am. J. Physiol. Heart Circ. Physiol.* 310 1–30. 10.1152/ajpheart.00832.2015 26873966 PMC4867337

[B123] Pillai V. B. Kanwal A. Fang Y. H. Sharp W. W. Samant S. Arbiser J. (2017). Honokiol, an activator of Sirtuin-3 (SIRT3) preserves mitochondria and protects the heart from doxorubicin-induced cardiomyopathy in mice. *Oncotarget* 8 34082–34098. 10.18632/oncotarget.16133 28423723 PMC5470953

[B124] Pillai V. B. Samant S. Sundaresan N. R. Raghuraman H. Kim G. Bonner M. Y. (2015). Honokiol blocks and reverses cardiac hypertrophy in mice by activating mitochondrial Sirt3. *Nat. Commun.* 6:6656. 10.1038/ncomms7656 25871545 PMC4441304

[B125] Pillai V. B. Sundaresan N. R. Kim G. Gupta M. Rajamohan S. B. Pillai J. B. (2010). Exogenous NAD blocks cardiac hypertrophic response via activation of the SIRT3-LKB1-AMP-activated kinase pathway. *J. Biol. Chem.* 285 3133–3144. 10.1074/jbc.M109.077271 19940131 PMC2823454

[B126] Planavila A. Iglesias R. Giralt M. Villarroya F. (2011). Sirt1 acts in association with PPARα to protect the heart from hypertrophy, metabolic dysregulation, and inflammation. *Cardiovasc. Res.* 90 276–284. 10.1093/cvr/cvq376 21115502

[B127] Porter G. A. Urciuoli W. R. Brookes P. S. Nadtochiy S. M. (2014). SIRT3 deficiency exacerbates ischemia-reperfusion injury: implication for aged hearts. *Am. J. Physiol. Heart Circ. Physiol.* 306 H1602–H1609. 10.1152/ajpheart.00027.2014 24748594 PMC4059981

[B128] Qiu X. Brown K. Hirschey M. D. Verdin E. Chen D. (2010). Calorie restriction reduces oxidative stress by SIRT3-mediated SOD2 activation. *Cell Metab.* 12 662–667. 10.1016/j.cmet.2010.11.015 21109198

[B129] Rahn J. J. Stackley K. D. Chan S. S. L. (2013). Opa1 is required for proper mitochondrial metabolism in early development. *PLoS One* 8:e59218. 10.1371/journal.pone.0059218 23516612 PMC3597633

[B130] Rardin M. J. He W. Nishida Y. Newman J. C. Carrico C. Danielson S. R. (2013a). SIRT5 regulates the mitochondrial lysine succinylome and metabolic networks. *Cell Metab.* 18 920–933. 10.1016/j.cmet.2013.11.013 24315375 PMC4105152

[B131] Rardin M. J. Newman J. C. Held J. M. Cusack M. P. Sorensen D. J. Li B. (2013b). Label-free quantitative proteomics of the lysine acetylome in mitochondria identifies substrates of SIRT3 in metabolic pathways. *Proc. Natl. Acad. Sci. U.S.A.* 110 6601–6606. 10.1073/pnas.1302961110 23576753 PMC3631688

[B132] Rose G. Dato S. Altomare K. Bellizzi D. Garasto S. Greco V. (2003). Variability of the SIRT3 gene, human silent information regulator Sir2 homologue, and survivorship in the elderly. *Exp. Gerontol.* 38 1065–1070. 10.1016/S0531-5565(03)00209-2 14580859

[B133] Ryu D. Jo Y. S. Sasso Lo G. Stein S. Zhang H. Perino A. (2014). A SIRT7-dependent acetylation switch of GABPβ1 controls mitochondrial function. *Cell Metab.* 20 856–869. 10.1016/j.cmet.2014.08.001 25200183

[B134] Saito T. Sadoshima J. (2015). Molecular mechanisms of mitochondrial autophagy/mitophagy in the heart. *Circ. Res.* 116 1477–1490. 10.1161/CIRCRESAHA.116.303790 25858070 PMC4419704

[B135] Samant S. A. Zhang H. J. Hong Z. Pillai V. B. Sundaresan N. R. Wolfgeher D. (2014). SIRT3 deacetylates and activates OPA1 to regulate mitochondrial dynamics during stress. *Mol. Cell. Biol.* 34 807–819. 10.1128/MCB.01483-13 24344202 PMC4023816

[B136] Schlicker C. Gertz M. Papatheodorou P. Kachholz B. Becker C. F. W. Steegborn C. (2008). Substrates and regulation mechanisms for the human mitochondrial sirtuins Sirt3 and Sirt5. *J. Mol. Biol.* 382 790–801. 10.1016/j.jmb.2008.07.048 18680753

[B137] Schrepfer E. Scorrano L. (2016). Mitofusins, from mitochondria to metabolism. *Mol. Cell* 61 683–694. 10.1016/j.molcel.2016.02.022 26942673

[B138] Schwer B. Bunkenborg J. Verdin R. O. Andersen J. S. Verdin E. (2006). Reversible lysine acetylation controls the activity of the mitochondrial enzyme acetyl-CoA synthetase 2. *Proc. Natl. Acad. Sci. U.S.A.* 103 10224–10229. 10.1073/pnas.0603968103 16788062 PMC1502439

[B139] Schwer B. Eckersdorff M. Li Y. Silva J. C. Fermin D. Kurtev M. V. (2009). Calorie restriction alters mitochondrial protein acetylation. *Aging Cell* 8 604–606. 10.1111/j.1474-9726.2009.00503.x 19594485 PMC2752488

[B140] Scott I. Webster B. R. Li J. H. Sack M. N. (2012). Identification of a molecular component of the mitochondrial acetyltransferase programme: a novel role for GCN5L1. *Biochem. J.* 443 655–661. 10.1042/BJ20120118 22309213 PMC7461726

[B141] Sebastiani M. Giordano C. Nediani C. Travaglini C. Borchi E. Zani M. (2007). Induction of mitochondrial biogenesis is a maladaptive mechanism in mitochondrial cardiomyopathies. *J. Am. Coll. Cardiol.* 50 1362–1369. 10.1016/j.jacc.2007.06.035 17903636

[B142] Shi G. McQuibban G. A. (2017). The mitochondrial rhomboid protease PARL is regulated by PDK2 to integrate mitochondrial quality control and metabolism. *Cell Rep.* 18 1458–1472. 10.1016/j.celrep.2017.01.029 28178523

[B143] Shi T. Wang F. Stieren E. Tong Q. (2005). SIRT3, a mitochondrial sirtuin deacetylase, regulates mitochondrial function and thermogenesis in brown adipocytes. *J. Biol. Chem.* 280 13560–13567. 10.1074/jbc.M414670200 15653680

[B144] Shiau M.-Y. Lee P.-S. Huang Y.-J. Yang C.-P. Hsiao C.-W. Chang K.-Y. (2017). Role of PARL-PINK1-Parkin pathway in adipocyte differentiation. *Metab. Clin. Exp.* 72 1–17. 10.1016/j.metabol.2017.03.010 28641777

[B145] Shinmura K. Tamaki K. Sano M. Nakashima-Kamimura N. Wolf A. M. Amo T. (2011). Caloric restriction primes mitochondria for ischemic stress by deacetylating specific mitochondrial proteins of the electron transport chain. *Circ. Res.* 109 396–406. 10.1161/CIRCRESAHA.111.243097 21700931

[B146] Signorile A. Santeramo A. Tamma G. Pellegrino T. D’Oria S. Lattanzio P. (2017). Mitochondrial cAMP prevents apoptosis modulating Sirt3 protein level and OPA1 processing in cardiac myoblast cells. *Biochim. Biophys. Acta* 1864 355–366. 10.1016/j.bbamcr.2016.11.022 27890624

[B147] Sol E. M. Wagner S. A. Weinert B. T. Kumar A. Kim H. S. Deng C.-X. (2012). Proteomic investigations of lysine acetylation identify diverse substrates of mitochondrial deacetylase Sirt3. *PLoS One* 7:e50545. 10.1371/journal.pone.0050545 23236377 PMC3517600

[B148] Someya S. Yu W. Hallows W. C. Xu J. Vann J. M. Leeuwenburgh C. (2010). Sirt3 mediates reduction of oxidative damage and prevention of age-related hearing loss under caloric restriction. *Cell* 143 802–812. 10.1016/j.cell.2010.10.002 21094524 PMC3018849

[B149] Song M. Franco A. Fleischer J. A. Zhang L. Dorn G. W. (2017). Abrogating mitochondrial dynamics in mouse hearts accelerates mitochondrial senescence. *Cell Metab.* 26 872.e5–883.e5. 10.1016/j.cmet.2017.09.023 29107503 PMC5718956

[B150] Sultana M. R. Bagul P. K. Katare P. B. Anwar Mohammed S. Padiya R. Banerjee S. K. (2016). Garlic activates SIRT-3 to prevent cardiac oxidative stress and mitochondrial dysfunction in diabetes. *Life Sci.* 164 42–51. 10.1016/j.lfs.2016.08.030 27590611

[B151] Sundaresan N. R. Bindu S. Pillai V. B. Samant S. Pan Y. Huang J.-Y. (2016). SIRT3 blocks aging-associated tissue fibrosis in mice by deacetylating and activating glycogen synthase kinase 3β. *Mol. Cell. Biol.* 36 678–692. 10.1128/MCB.00586-15 26667039 PMC4760222

[B152] Sundaresan N. R. Gupta M. Kim G. Rajamohan S. B. Isbatan A. Gupta M. P. (2009). Sirt3 blocks the cardiac hypertrophic response by augmenting Foxo3a-dependent antioxidant defense mechanisms in mice. *J. Clin. Invest.* 119 2758–2771. 10.1172/JCI39162 19652361 PMC2735933

[B153] Sundaresan N. R. Samant S. A. Pillai V. B. Rajamohan S. B. Gupta M. P. (2008). SIRT3 is a stress-responsive deacetylase in cardiomyocytes that protects cells from stress-mediated cell death by deacetylation of Ku70. *Mol. Cell. Biol.* 28 6384–6401. 10.1128/MCB.00426-08 18710944 PMC2577434

[B154] Sundaresan N. R. Vasudevan P. Zhong L. Kim G. Samant S. Parekh V. (2012). The sirtuin SIRT6 blocks IGF-Akt signaling and development of cardiac hypertrophy by targeting c-Jun. *Nat. Med.* 18 1643–1650. 10.1038/nm.2961 23086477 PMC4401084

[B155] Taherzadeh-Fard E. Saft C. Akkad D. A. Wieczorek S. Haghikia A. Chan A. (2011). PGC-1alpha downstream transcription factors NRF-1 and TFAM are genetic modifiers of Huntington disease. *Mol. Neurodegener.* 6:32. 10.1186/1750-1326-6-32 21595933 PMC3117738

[B156] Tan M. Peng C. Anderson K. A. Chhoy P. Xie Z. Dai L. (2014). Lysine glutarylation is a protein posttranslational modification regulated by SIRT5. *Cell Metab.* 19 605–617. 10.1016/j.cmet.2014.03.014 24703693 PMC4108075

[B157] Tang X. Chen X.-F. Wang N.-Y. Wang X.-M. Liang S.-T. Zheng W. (2017). SIRT2 acts as a cardioprotective deacetylase in pathological cardiac hypertrophy. *Circulation* 136 2051–2067. 10.1161/CIRCULATIONAHA.117.028728 28947430 PMC5698109

[B158] Tang Y. Mi C. Liu J. Gao F. Long J. (2014). Compromised mitochondrial remodeling in compensatory hypertrophied myocardium of spontaneously hypertensive rat. *Cardiovasc. Pathol.* 23 101–106. 10.1016/j.carpath.2013.11.002 24388463

[B159] Tanno M. Kuno A. Yano T. Miura T. Hisahara S. Ishikawa S. (2010). Induction of manganese superoxide dismutase by nuclear translocation and activation of SIRT1 promotes cell survival in chronic heart failure. *J. Biol. Chem.* 285 8375–8382. 10.1074/jbc.M109.090266 20089851 PMC2832987

[B160] Tanno M. Sakamoto J. Miura T. Shimamoto K. Horio Y. (2007). Nucleocytoplasmic shuttling of the NAD+-dependent histone deacetylase SIRT1. *J. Biol. Chem.* 282 6823–6832. 10.1074/jbc.M609554200 17197703

[B161] Tao R. Coleman M. C. Pennington J. D. Ozden O. Park S. H. Jiang H. (2010). Sirt3-mediated deacetylation of evolutionarily conserved lysine 122 regulates MnSOD activity in response to stress. *Mol. Cell* 40 893–904. 10.1016/j.molcel.2010.12.013 21172655 PMC3266626

[B162] Teng Y.-B. Jing H. Aramsangtienchai P. He B. Khan S. Hu J. (2015). Efficient demyristoylase activity of SIRT2 revealed by kinetic and structural studies. *Sci. Rep.* 5:8529. 10.1038/srep08529 25704306 PMC4894398

[B163] Thapa D. Zhang M. Manning J. R. Guimarães D. A. Stoner M. W. O’Doherty R. M. (2017). Acetylation of mitochondrial proteins by GCN5L1 promotes enhanced fatty acid oxidation in the heart. *Am. J. Physiol. Heart Circ. Physiol.* 313 H265–H274. 10.1152/ajpheart.00752.2016 28526709 PMC5582919

[B164] Tong Z. Wang M. Wang Y. Kim D. D. Grenier J. K. Cao J. (2017). SIRT7 is an RNA-activated protein lysine deacylase. *ACS Chem. Biol.* 12 300–310. 10.1021/acschembio.6b00954 27997115 PMC5326686

[B165] Tong Z. Wang Y. Zhang X. Kim D. D. Sadhukhan S. Hao Q. (2016). SIRT7 is activated by DNA and deacetylates histone H3 in the chromatin context. *ACS Chem. Biol.* 11 742–747. 10.1021/acschembio.5b01084 26907567 PMC4850736

[B166] Tseng A. H. H. Shieh S.-S. Wang D. L. (2013). SIRT3 deacetylates FOXO3 to protect mitochondria against oxidative damage. *Free Radic. Biol. Med.* 63 222–234. 10.1016/j.freeradbiomed.2013.05.002 23665396

[B167] Vakhrusheva O. Smolka C. Gajawada P. Kostin S. Boettger T. Kubin T. (2008). Sirt7 increases stress resistance of cardiomyocytes and prevents apoptosis and inflammatory cardiomyopathy in mice. *Circ. Res.* 102 703–710. 10.1161/CIRCRESAHA.107.164558 18239138

[B168] van der Bliek A. M. Shen Q. Kawajiri S. (2013). Mechanisms of mitochondrial fission and fusion. *Cold Spring Harb. Perspect. Biol.* 5:a011072. 10.1101/cshperspect.a011072 23732471 PMC3660830

[B169] Vaquero A. Scher M. B. Lee D. H. Sutton A. Cheng H. L. Alt F. W. (2006). SirT2 is a histone deacetylase with preference for histone H4 Lys 16 during mitosis. *Genes Dev.* 20 1256–1261. 10.1101/gad.1412706 16648462 PMC1472900

[B170] Vassilopoulos A. Pennington J. D. Andresson T. Rees D. M. Bosley A. D. Fearnley I. M. (2014). SIRT3 deacetylates ATP synthase F1 complex proteins in response to nutrient- and exercise-induced stress. *Antioxid. Redox Signal.* 21 551–564. 10.1089/ars.2013.5420 24252090 PMC4085980

[B171] Vaziri H. Dessain S. K. Ng Eaton E. Imai S. I. Frye R. A. Pandita T. K. (2001). hSIR2(SIRT1) functions as an NAD-dependent p53 deacetylase. *Cell* 107 149–159. 10.1038/35106033 11672523

[B172] Wagner G. R. Pride P. M. Babbey C. M. Payne R. M. (2012). Friedreich’s ataxia reveals a mechanism for coordinate regulation of oxidative metabolism via feedback inhibition of the SIRT3 deacetylase. *Hum. Mol. Genet.* 21 2688–2697. 10.1093/hmg/dds095 22394676 PMC3363336

[B173] Wang G. Han T. Nijhawan D. Theodoropoulos P. Naidoo J. Yadavalli S. (2014). P7C3 neuroprotective chemicals function by activating the rate-limiting enzyme in NAD salvage. *Cell* 158 1324–1334. 10.1016/j.cell.2014.07.040 25215490 PMC4163014

[B174] Wang X. Hu S. Liu L. (2017). Phosphorylation and acetylation modifications of FOXO3a: Independently or synergistically? *Oncol. Lett.* 13 2867–2872. 10.3892/ol.2017.5851 28521392 PMC5431355

[B175] Watanabe H. Inaba Y. Kimura K. Matsumoto M. Kaneko S. Kasuga M. (2017). Sirt2 facilitates hepatic glucose uptake by deacetylating glucokinase regulatory protein. *Nat. Commun.* 9 1–14. 10.1038/s41467-017-02537-6 29296001 PMC5750207

[B176] Wei T. Huang G. Gao J. Huang C. Sun M. Wu J. (2017). Sirtuin 3 deficiency accelerates hypertensive cardiac remodeling by impairing angiogenesis. *J. Am. Heart Assoc.* 6 e6114–e6119. 10.1161/JAHA.117.006114 28862956 PMC5586452

[B177] Weinert B. T. Moustafa T. Iesmantavicius V. Zechner R. Choudhary C. (2015). Analysis of acetylation stoichiometry suggests that SIRT3 repairs nonenzymatic acetylation lesions. *EMBO J.* 34 2620–2632. 10.15252/embj.201591271 26358839 PMC4641529

[B178] Wende A. R. O’Neill B. T. Bugger H. Riehle C. Tuinei J. Buchanan J. (2015). Enhanced cardiac Akt/protein kinase B signaling contributes to pathological cardiac hypertrophy in part by impairing mitochondrial function via transcriptional repression of mitochondrion-targeted nuclear genes. *Mol. Cell. Biol.* 35 831–846. 10.1128/MCB.01109-14 25535334 PMC4323486

[B179] Wu Y.-T. Lee H.-C. Liao C.-C. Wei Y.-H. (2013). Regulation of mitochondrial F(o)F(1)ATPase activity by Sirt3-catalyzed deacetylation and its deficiency in human cells harboring 4977bp deletion of mitochondrial DNA. *Biochim. Biophys. Acta* 1832 216–227. 10.1016/j.bbadis.2012.10.002 23046812

[B180] Yang B. Zwaans B. M. M. Eckersdorff M. Lombard D. B. (2009). The sirtuin SIRT6 deacetylates H3 K56Ac in vivo to promote genomic stability. *Cell Cycle* 8 2662–2663. 10.4161/cc.8.16.9329 19597350 PMC2728171

[B181] Yang H. Yang T. Baur J. A. Perez E. Matsui T. Carmona J. J. (2007). Nutrient-sensitive mitochondrial NAD+ levels dictate cell survival. *Cell* 130 1095–1107. 10.1016/j.cell.2007.07.035 17889652 PMC3366687

[B182] Yang W. Nagasawa K. Münch C. Xu Y. Satterstrom K. Jeong S. (2016a). Mitochondrial sirtuin network reveals dynamic SIRT3-dependent deacetylation in response to membrane depolarization. *Cell* 167 985.e–1000.e. 10.1016/j.cell.2016.10.016 27881304 PMC5134900

[B183] Yang Y. Wang W. Xiong Z. Kong J. Qiu Y. Shen F. (2016b). Activation of SIRT3 attenuates triptolide-induced toxicity through closing mitochondrial permeability transition pore in cardiomyocytes. *Toxicol. In Vitro* 34 128–137. 10.1016/j.tiv.2016.03.020 27064125

[B184] Yang Y. Cimen H. Han M. J. Shi T. Deng J. H. Koc H. (2010). NAD+-dependent deacetylase SIRT3 regulates mitochondrial protein synthesis by deacetylation of the ribosomal protein MRPL10. *J. Biol. Chem.* 285 7417–7429. 10.1074/jbc.M109.053421 20042612 PMC2844190

[B185] Yoshino J. Mills K. F. Yoon M. J. Imai S.-I. (2011). Nicotinamide mononucleotide, a key NAD(+) intermediate, treats the pathophysiology of diet- and age-induced diabetes in mice. *Cell Metab.* 14 528–536. 10.1016/j.cmet.2011.08.014 21982712 PMC3204926

[B186] Yu W. Dittenhafer-Reed K. E. Denu J. M. (2012). SIRT3 protein deacetylates isocitrate dehydrogenase 2 (IDH2) and regulates mitochondrial redox status. *J. Biol. Chem.* 287 14078–14086. 10.1074/jbc.M112.355206 22416140 PMC3340192

[B187] Yu W. Gao B. Li N. Wang J. Qiu C. Zhang G. (2017). Sirt3 deficiency exacerbates diabetic cardiac dysfunction: role of Foxo3A-Parkin-mediated mitophagy. *Biochim. Biophys. Acta* 1863 1973–1983. 10.1016/j.bbadis.2016.10.021 27794418

[B188] Zhang X. Ji R. Liao X. Castillero E. Kennel P. J. Brunjes D. L. (2018). miR-195 regulates metabolism in failing myocardium via alterations in SIRT3 expression and mitochondrial protein acetylation. *Circulation* 137 2052–2067. 10.1161/CIRCULATIONAHA.117.030486 29330215 PMC6449058

[B189] Zhang Y. Wang B. Fu X. Guan S. Han W. Zhang J. (2016). Exogenous NAD+administration significantly protects against myocardial ischemia/reperfusion injury in rat model. *Am. J. Transl. Res.* 8 3342–3350. 27648125 PMC5009387

[B190] Zhao S. Xu W. Jiang W. Yu W. Lin Y. Zhang T. (2010). Regulation of cellular metabolism by protein lysine acetylation. *Science* 327 1000–1004. 10.1126/science.1179689 20167786 PMC3232675

[B191] Zhu J. Vinothkumar K. R. Hirst J. (2016). Structure of mammalian respiratory complex I. *Nature* 536 354–358. 10.1038/nature19095 27509854 PMC5027920

